# Overexpression of zinc finger DHHC-type containing 1 is associated with poor prognosis and cancer cell growth and metastasis in uterine corpus endometrial carcinoma

**DOI:** 10.18632/aging.205899

**Published:** 2024-06-06

**Authors:** Ni Jiang, Dan Li, Ye Han, Zhi-Guo Luo, Lu-Bin Liu

**Affiliations:** 1 Department of Obstetrics and Gynecology, Women and Children’s Hospital of Chongqing Medical University, Chongqing, China; 2 Department of Oncology, Taihe Hospital, Hubei University of Medicine, Shiyan, China

**Keywords:** ZDHHC1, UCEC, nomogram, immune infiltration, RNA modification

## Abstract

The zinc finger DHHC-type containing 1 (ZDHHC1) gene is implicated in the pathogenesis and progression of various malignant tumors, but its precise involvement in uterine corpus endometrial carcinoma (UCEC) remains unknown. Thus, this study investigated ZDHHC1 expression in UCEC using publicly available TCGA and Xena databases and elucidated the functions and mechanisms of the ZDHHC1 gene in UCEC progression using bioinformatics and *in vitro* experiments. The correlation between ZDHHC1 expression and prognosis, clinical features, immune cells, and RNA modifications of UCEC was evaluated using nomograms, correlation, ROC, and survival analyses. The impacts of ZDHHC1 overexpression on UCEC progression and mechanisms were explored with bioinformatics and *in vitro* experiments. Our study revealed that ZDHHC1 expression was significantly downregulated in UCEC and correlated with poor prognosis, cancer diagnosis, clinical stage, age, weight, body mass index, histological subtypes, residual tumor, tumor grade, and tumor invasion. Notably, Cox regression analysis and constructed nomograms showed that downregulated ZDHHC1 expression was a prognostic factor associated with poor prognosis in patients with UCEC. Conversely, above-normal ZDHHC1 expression inhibited the cell growth, cell cycle transition, migration, and invasion of UCEC cells, which may be related to the cell cycle, DNA replication, PI3K-AKT, and other pathways that promote tumor progression. Altered ZDHHC1 expression in UCEC was significantly associated with RNA modifications and the changes in cancer immune cell populations, such as CD56 bright NK cells, eosinophils, Th2 cells, and cell markers. In conclusion, considerably reduced ZDHHC1 expression in UCEC is associated with cancer cell growth, metastasis, poor prognosis, immune infiltration, and RNA modifications, revealing the promising potential of ZDHHC1 as a prognostic marker for UCEC.

## INTRODUCTION

Endometrial carcinoma is a prevalent gynecological malignant tumor with high incidence and mortality rates in recent decades [1]. While various treatments exist, the prognosis for patients with advanced-stage endometrial carcinoma remains suboptimal compared to those with early-stage diagnosis, whose prognosis is favorable. Several treatment targets play a critical role in endometrial carcinoma progression [2–4], including maternal embryonic leucine zipper kinase (MELK), an enzyme frequently overexpressed in endometrial carcinoma tissues. High MELK expression correlates with histological subtype, higher grade, advanced clinical stage, decreased overall survival (OS), and reduced disease-free survival (DFS) in endometrial carcinoma. Moreover, MELK overexpression promotes endometrial carcinoma progression by modulating the E2F transcription factor 1 (E2F1) protein and activating the mTORC1 (mammalian transducer of regulated cAMP response element-binding protein) and mTORC2 signaling pathways [2]. Conversely, downregulated MELK expression reduces proliferation, migration, and tumorigenesis in endometrial carcinoma cells [2]. Since targeted therapy shows great promise in improving the prognosis of endometrial carcinoma [1, 5], identifying novel therapeutic targets is critical in combating the disease.

The zinc finger DHHC-type containing 1 (ZDHHC1) protein correlates significantly with the occurrence and development of tumor and non-tumor diseases [6–10]. For example, Wang et al. [6] found that the tumor suppressor p53 gene represses the replication of Japanese encephalitis virus by upregulating interferon-induced transmembrane protein 3 (IFITM3). The p53 gene induces IFITM3 protein expression by enhancing ZDHHC1-mediated IFITM3 palmitoylation, which upregulates ZDHHC1/IFITM3 signaling pathway and inhibits viral replication [6]. Le et al. reported that ZDHHC1 expression is silenced in tumor cells and tissues, and its recovery inhibits cancer progression via various mechanisms, such as stimulating cell apoptosis, inducing cell cycle arrest, inhibiting cell metastasis, and reversing epithelial-mesenchymal transition and cell dryness. They also observed that ZDHHC1 negatively regulates the metabolism of tumor cells and stimulates oxidative and endoplasmic reticulum stress to induce apoptosis [8]. Although the ZDHHC1 protein is implicated in the pathogenesis and progression of various cancers, its roles and mechanisms in uterine corpus endometrial carcinoma (UCEC) are unknown. Therefore, we used bioinformatics and *in vitro* experiments to explore ZDHHC1 roles and mechanisms underlying UCEC progression and how ZDHHC1 expression correlates with UCEC prognosis.

## MATERIALS AND METHODS

### Data sources

Transcript per million (TPM)-quantified gene expression data of patients with UCEC were retrieved on October 2022 from The Cancer Genome Atlas (TCGA), Xena TCGA, and Genotype-Tissue Expression (GTEx) databases. On the same day, the prognosis and clinical data of patients with UCEC were downloaded from TCGA database. Data from TCGA database consisted of 35 normal endometrial and 554 UCEC tissue samples, of which 23 normal endometrial and 23 cancer tissues had identical origins. The Xena-TCGA database included 23 normal endometrial and 181 UCEC tissue samples, while the GTEx database had 78 normal endometrial samples.

### Identification of ZDHHC1 expression in UCEC tissues

We performed an expression analysis of unmatched and paired normal and UCEC tissues to explore ZDHHC1 expression in UCEC. We also matched ZDHHC1 expression data with clinical data of patients with UCEC and eliminated any missing information. Subsequently, we grouped the patients according to their clinical characteristics and examined the expression levels of ZDHHC1 in UCEC tissues.

### Clinical roles of ZDHHC1 expression

We performed a receiver operating characteristic (ROC) analysis to assess the diagnostic value of ZDHHC1 in detecting UCEC, with the area under the curve (AUC) as the evaluation standard [11]. We matched and sorted ZDHHC1 expression data with clinical data of patients with UCEC to explore the relationship between ZDHHC1 expression and prognostic and clinical characteristics of patients. We grouped the patients based on the median value of ZDHHC1 expression to investigate any correlation with such features.

### Identification of the relationship between decreased ZDHHC1 expression and prognosis of subgroup patients with UCEC

The relationship between ZDHHC1 expression levels and the prognosis of a subgroup of patients with UCEC was investigated using the prognostic and clinical data of patients with UCEC from the TCGA database. The patients were grouped based on the median value of ZDHHC1 expression. The association between the changes in ZDHHC1 expression and the prognostic indicators of the patient subgroup was evaluated using Kaplan-Meier survival analysis, with *P* < 0.05 as the criterion for statistical significance.

### ZDHHC1-related nomograms

Univariate Cox analysis was employed to identify the relationship between ZDHHC1 expression or various clinical factors (such as the clinical stage, age, weight, height, BMI, histological subtype, tumor status, tumor invasion, hormone therapy, and radiotherapy) and the survival outcome of patients with UCEC. We considered the *P*-value of the statistical indicator to establish the prognostic nomograms and estimated the 1-year, 3-year, and 5-year survival of patients.

### Visualization of functional mechanisms and network of ZDHHC1-related genes

Spearman rank correlation was used to recognize genes whose expression strongly correlated with ZDHHC1 expression in UCEC tissues. Genes were considered strongly correlated if the absolute value of the correlation coefficient was greater than 0.4 and *P* < 0.001. The GO and KEGG analyses of 825 ZDHHC1-related genes were performed using the DAVID database to investigate their functions and pathways. A protein network of ZDHHC1-related genes was established using the STRING database [12] and visualized with Cytoscape.

### Construction of ZDHHC1-overexpressing UCEC cells

Endometrial carcinoma Ishikawa cells and endometrial adenosquamous carcinoma RL95-2 cells were purchased from the Typical Culture Collection Center in the United States or the European Collection Center. Ishikawa cells were cultured in Minimum Essential Medium (MEM) containing 5% fetal bovine serum, and RL95-2 cells were cultured in Dulbecco’s modified Eagle medium/Nutrient Mixture F-12 (DMEM/F-12) medium containing 10% fetal bovine serum. The culture conditions for all cells were 5% CO_2_ and 37°C. The ZDHHC1 overexpression (ZDHHC1-OV) and empty control (Control) vectors were purchased from Youbao Biotechnology. Ishiwaka and RL95-2 cells were transfected with the vectors following the manufacturer’s instructions. The target gene sequence of ZDHHC1 was ENSG0000159714.

### Validation of UCEC cell models

Cells were lysed on ice, and total RNA or proteins were extracted from the control and ZDHHC1 overexpression cells. Total RNA was isolated and quantified, followed by reverse transcription and PCR amplification. The concentration of the extracted proteins was calculated with the bicinchoninic acid method, and the proteins were separated using gel electrophoresis. Incubation was done with primary and secondary antibodies. The relative expression levels of ZDHHC1 in control and ZDHHC1 overexpression cells were calculated. The primer sequences for ZDHHC1 amplification were purchased from GeneCopoeia, Inc. (No. HQP099884). The concentrations of antibodies against ZDHHC1 and GAPDH were 1:1000, respectively. All experiments were repeated 3 times, with *P* < 0.05 as the criterion for determining statistical significance.

### Cell counting kit-8 (CCK-8) assay

The transfected suspended UCEC cells were collected, and the supernatant was discarded. After adjusting the cell density, cells were seeded onto 96-well plates, and each experimental group of cells occupied 3 wells, each with 3000 cells/well. Each well was filled with 10 ul of CCK-8 detection solution, and plates were placed in a humidified incubator for 2 h. The absorbance was measured at 450 nm in a microplate reader. The cells were monitored for 5 days, and the corresponding cell growth curve was generated. All experiments were repeated 3 times, with *P* < 0.05 as the criterion for assessing statistical significance.

### 5-ethynyl-2′-deoxyuridine (EdU) assay

The transfected UCEC cells were collected and cultured under appropriate temperature, humidity, and oxygen conditions. Cell culture medium was mixed with EdU solution, and cells were incubated for progressive EdU labeling. The medium was discarded, and the cells were fixed with 4% paraformaldehyde. Permeabilization with 0.5% Triton X-100 isotonic solution was performed to facilitate EdU entry to the nucleus. The results of EdU staining were observed under a fluorescence microscope. The proportion of EdU-positive cells and cell proliferation rate was calculated. All experiments were repeated 3 times, with *P* < 0.05 as the cutoff for calculating statistical significance.

### Cell cycle analysis by flow cytometry

The transfected UCEC cells were collected and cultured under appropriate temperature, humidity, and oxygen conditions. The cultured cells were fixed in 70% ethanol and refrigerated for 1 h. After removing cell fixatives, cells were stained with 50 μg/ml PI and 100 μg/ml RNase A phosphate-buffered saline. Cells were separated by flow cytometry, and the G0/G1, S, and G2/M phases were detected with flow cytometry software. All experiments were repeated 3 times, with *P* < 0.05 as the cutoff for determining statistical significance.

### Transwell assay

The transfected UCEC cells were collected and cultured under appropriate temperature, humidity, and oxygen conditions. After digestion and centrifugation, UCEC cells were resuspended with serum-free medium and gently blown evenly. Cell concentration was adjusted to 2 × 10^5^/ml. The upper chamber of Transwell plates was seeded with 200 μl of the cell suspension, and the lower one was filled with 600 μl of the medium. Transwell plates were placed in an incubator and incubated for 24 h. Cell migration through the permeable membrane was evaluated under a microscope. The culture medium in the upper chamber was poured out, and the residual cells on the upper chamber surface were removed with a cotton swab, followed by air drying. Cells were fixed in 4% paraformaldehyde, stained with 0.5% crystal violet for 5 min, photographed, and counted under the microscope. All experiments were repeated 3 times, with *P* < 0.05 as the cutoff for calculating statistical significance.

### Immune cell infiltration analysis

Tumor-infiltrating immune cell levels in UCEC tissues were quantified with single-sample gene set enrichment analysis (ssGSEA). The relationship between ZDHHC1 expression and tumor-infiltrating immune cell levels was explored using Spearman rank correlation. We divided the ZDHHC1 expression data into high-ZDHHC1 and low-ZDHHC1 expression groups based on the median value to investigate the statistical significance between the levels of tumor-infiltrating immune cells in the 2 groups. We extracted the expression data of immune cell markers from cancer tissues of patients with UCEC to explore the relationship between ZDHHC1 expression levels and immune cell markers using Spearman rank correlation. Statistical significance was inferred with a P threshold.

### Tumor immune estimation resource (TIMER) database

The relationship between gene expression and tumor-infiltrating immune cells or immune markers was assessed with the TIMER database [13]. The correlation between ZDHHC1 expression and the levels of tumor-infiltrating immune cells or cell markers in UCEC was verified using the correlation analysis module.

### Gene set enrichment analysis

Tumor development involves multiple signaling mechanisms [14, 15], and GSEA is a commonly used method to investigate the signaling mechanisms of individual or many genes involved in cancer progression [11, 16]. The ZDHHC1 gene was subjected to GSEA on the UCEC data from the TCGA database using the CAMOIP software. The analysis was conducted to explore the signaling mechanisms associated with ZDHHC1 expression levels in UCEC.

### The relationship between ZDHHC1 expression and RNA modifications

The relationship between genes and RNA modifications was investigated using the RM2Target database. Genes encoding RNA-modifying enzymes (hereafter RNA modification genes) related to ZDHHC1 were identified from the RM2TARGET database. Based on these findings, the correlation between ZDHHC1 and RNA modification gene expression was determined: ADAR, ADARB1, ALKBH1, ALKBH5, ALYREF, DKC1, ELAVL1, FBL, FTO, HNRNP2B1, HNRNPC, IGF2BP1, IGF2BP2, LRPPRC, METTL1, METTL14, METTL3, METTL5, NOP56, NOP58, PCIF1, PUS7, RBMX, WDR4, WTAP, YBX1, YTHDC1, YTHDC2, YTHDF1, YTHDF2, YTHDF3, ZC3H13, and ZCCHC4.

### Statistical analysis

The expression levels of ZDHHC1 in patients with UCEC and their subgroups were detected using a Wilcoxon rank sum test. The potential relationship between ZDHHC1 expression and UCEC diagnosis or prognosis was investigated using ROC and Kaplan-Meier survival analyses. In addition, the correlation between ZDHHC1 expression and immune microenvironment or RNA modifications was analyzed using Spearman rank correlation. Statistical significance was inferred when *P* < 0.05.

### Data availability statement

Data of patients investigated in this study are available at TCGA (https://www.cancer.gov/) and Xena (http://xena.ucsc.edu/) databases. The results of *in vitro* experiments can be obtained from the corresponding author upon request.

## RESULTS

### The expression of ZDHHC1 is downregulated in UCEC

**Figure 1:**
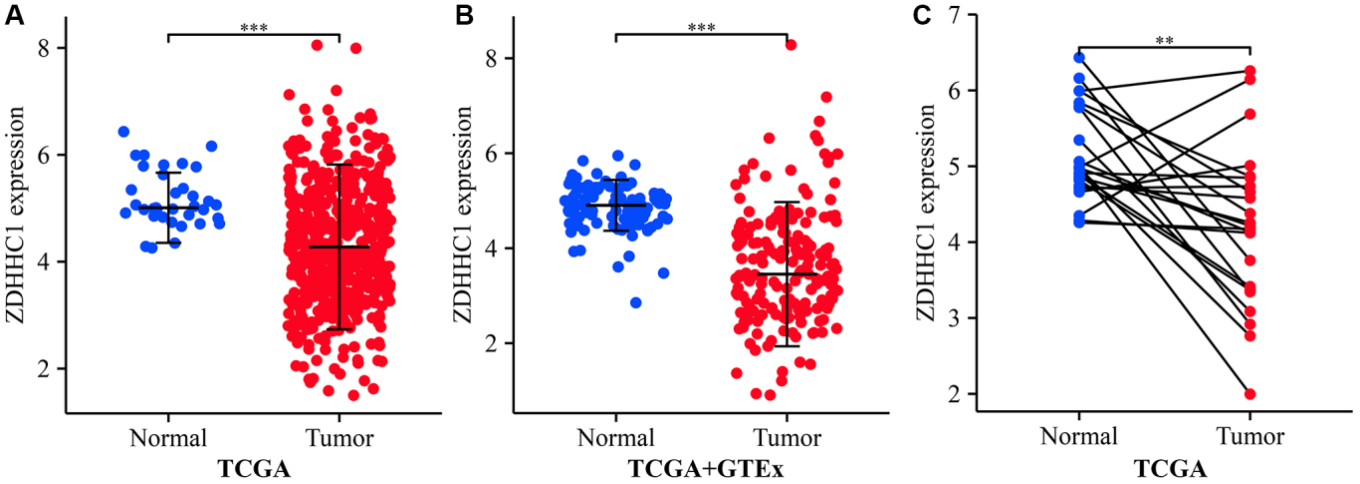
**ZDHHC1 expression in normal endometrial and UCEC tissues.** (**A**) TCGA database. (**B**) Xena database. (**C**) TCGA database. Abbreviations: UCEC: uterine corpus endometrial carcinoma; ZDHHC1: zinc finger DHHC-type containing 1; TCGA: The Cancer Genome Atlas; GTEx: Genotype-Tissue Expression.

**Figure 2:**
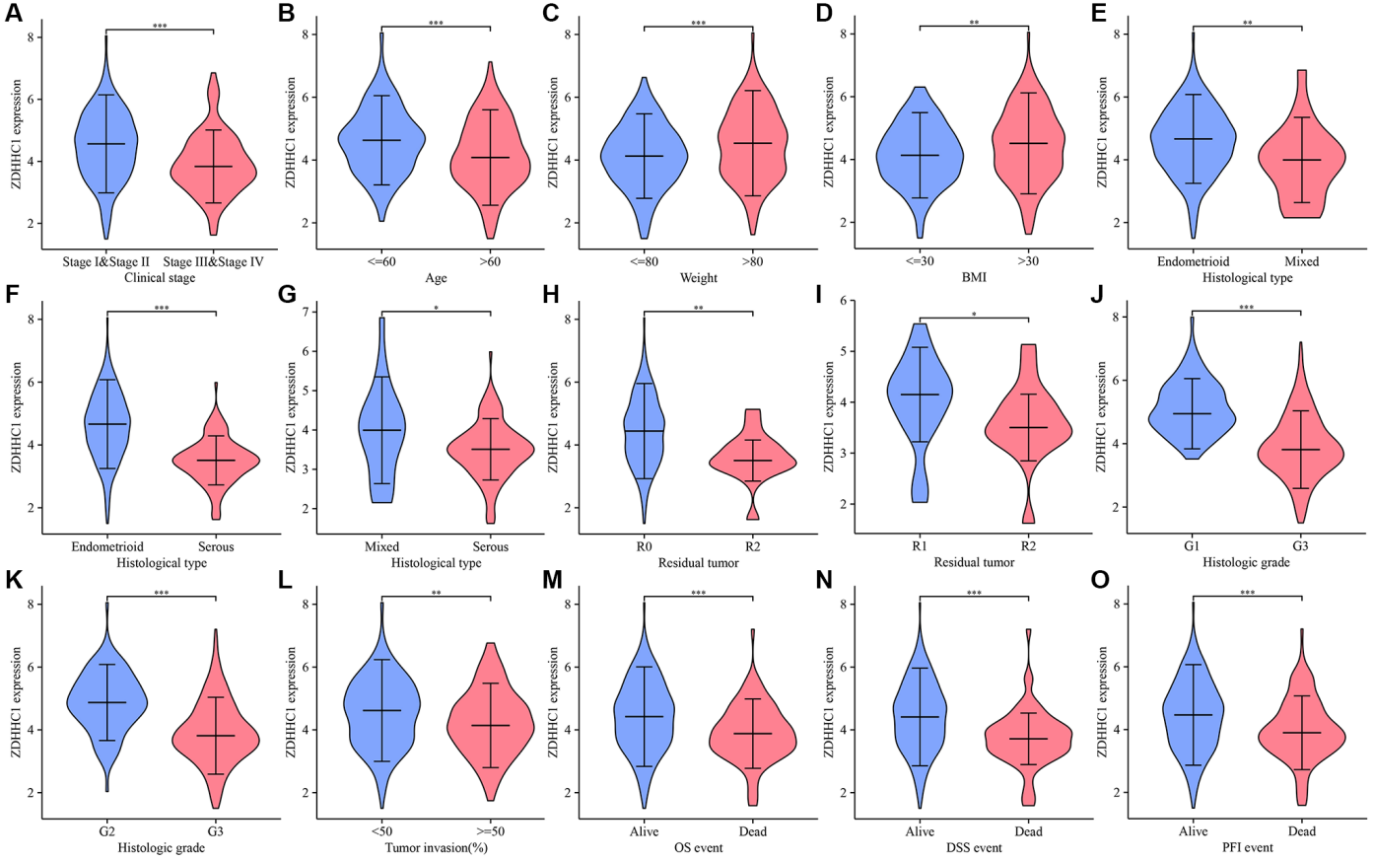
**ZDHHC1 expression in UCEC tissues derived from TCGA database.** (**A**) Tumor stage I–II vs. III–IV. (**B**) Age ≤60 vs. >60. (**C**) Weight ≤80 vs. >80. (**D**) BMI ≤30 vs. >30. (**E**) Endometrioid vs. mixed. (**F**) Endometrioid vs. serous. (**G**) Mixed vs. serous. (**H**) R0 vs. R2. (**I**) R1 vs. R2. (**J**) G1 vs. G3. (**K**) G2 vs. G3. (**L**) Tumor invasion (%) ≤50 vs. >50. (**M**–**O**) Alive vs. deceased. Abbreviations: UCEC: uterine corpus endometrial carcinoma; ZDHHC: zinc finger DHHC-type containing 1; TCGA: The Cancer Genome Atlas; BMI: body mass index; R0-R2: residual tumor; G1-G3: histopathologic grading.

Based on data from TCGA and Xena databases, ZDHHC1 expression was significantly lower in unpaired UCEC tissues than in normal endometrial tissues (Figure 1A, 1B). Similarly, ZDHHC1 levels were significantly reduced in 23 UCEC tissues versus their 23 matched normal endometrial tissues (Figure 1C). Additionally, ZDHHC1 levels were lower in deceased individuals and those with advanced age (>60), late clinical stage, serous subtype, and high histological grade. Conversely, ZDHHC1 levels were significantly higher in individuals with high body weight (>80 kg), high body mass index (BMI) (>30), and tumor invasion (<50) (Figure 2).

### Decreased ZDHHC1 expression is associated with UCEC diagnosis and a poor prognosis

**Figure 3:**
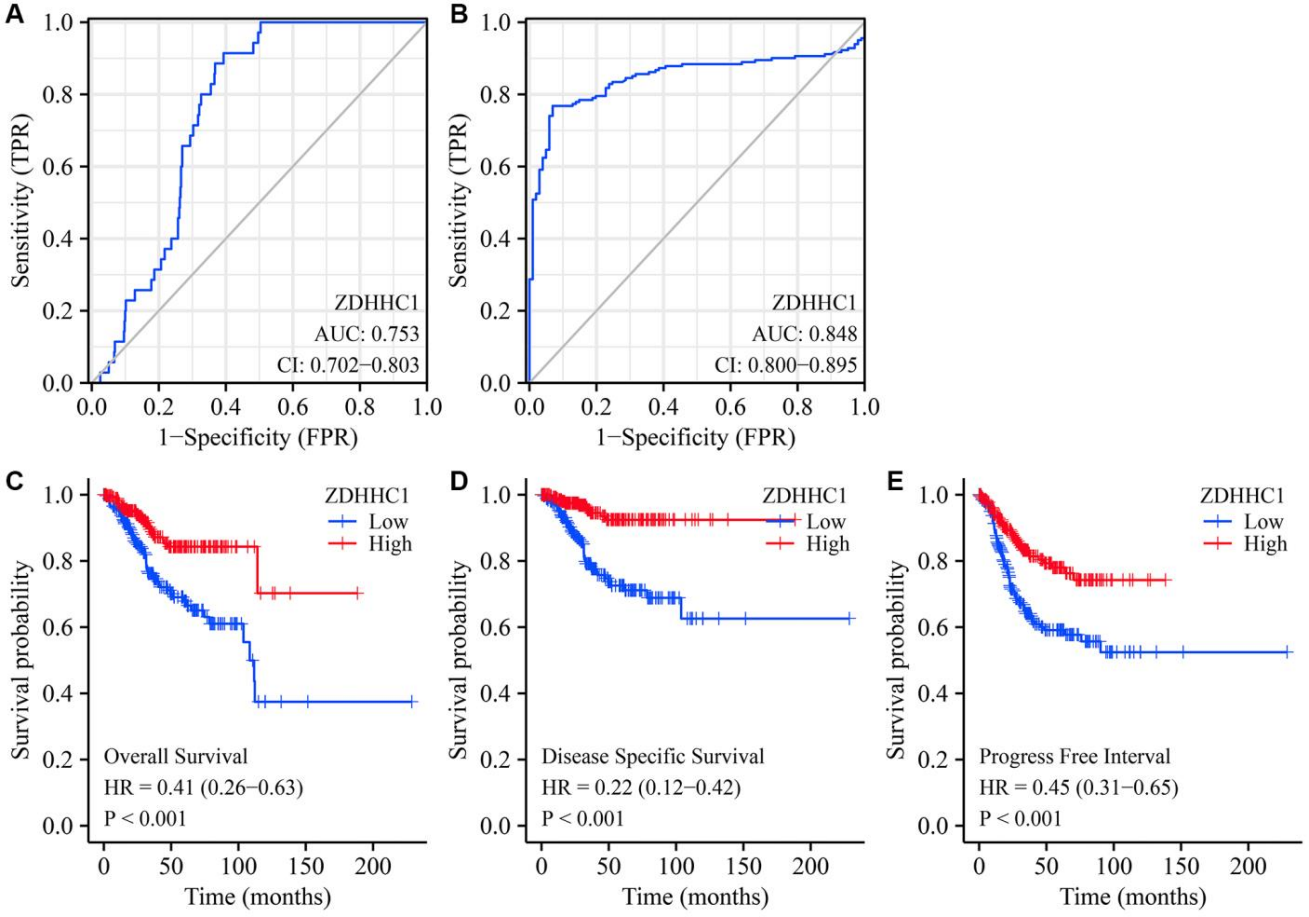
**ZDHHC1 expression is related to diagnosis and prognosis in UCEC.** (**A**, **B**) The diagnostic values of ZDHHC1 in UCEC obtained from TCGA and GTEx data in Xena database. (**C**–**E**) The prognostic values of ZDHHC1 in UCEC calculated from TCGA data. Abbreviations: UCEC: uterine corpus endometrial carcinoma; ZDHHC1: zinc finger DHHC-type containing 1; TCGA: The Cancer Genome Atlas; GTEx: Genotype-Tissue Expression.

The results of ROC analysis revealed that decreased ZDHHC1 expression was significantly indicative of diagnosing UCEC (Figure 3A, 3B). Specifically, when using the TCGA database, the AUC for ZDHHC1 was 0.753 (Figure 3A), and when also using the Xena database, the AUC was 0.848 (Figure 3B). Moreover, a Kaplan-Meier survival analysis demonstrated that decreased ZDHHC1 expression was a significant prognostic factor for UCEC (Figure 3C–3E). Specifically, reduced ZDHHC1 expression was associated with shorter OS (HR = 0.41; *P* < 0.001), disease-specific survival (DSS) (HR = 0.22; *P* < 0.001), and progression-free interval (PFI) (HR = 0.45; *P* < 0.001) in patients diagnosed with UCEC.

### Decreased ZDHHC1 expression is associated with poor prognosis in a subgroup of patients with UCEC

**Figure 4:**
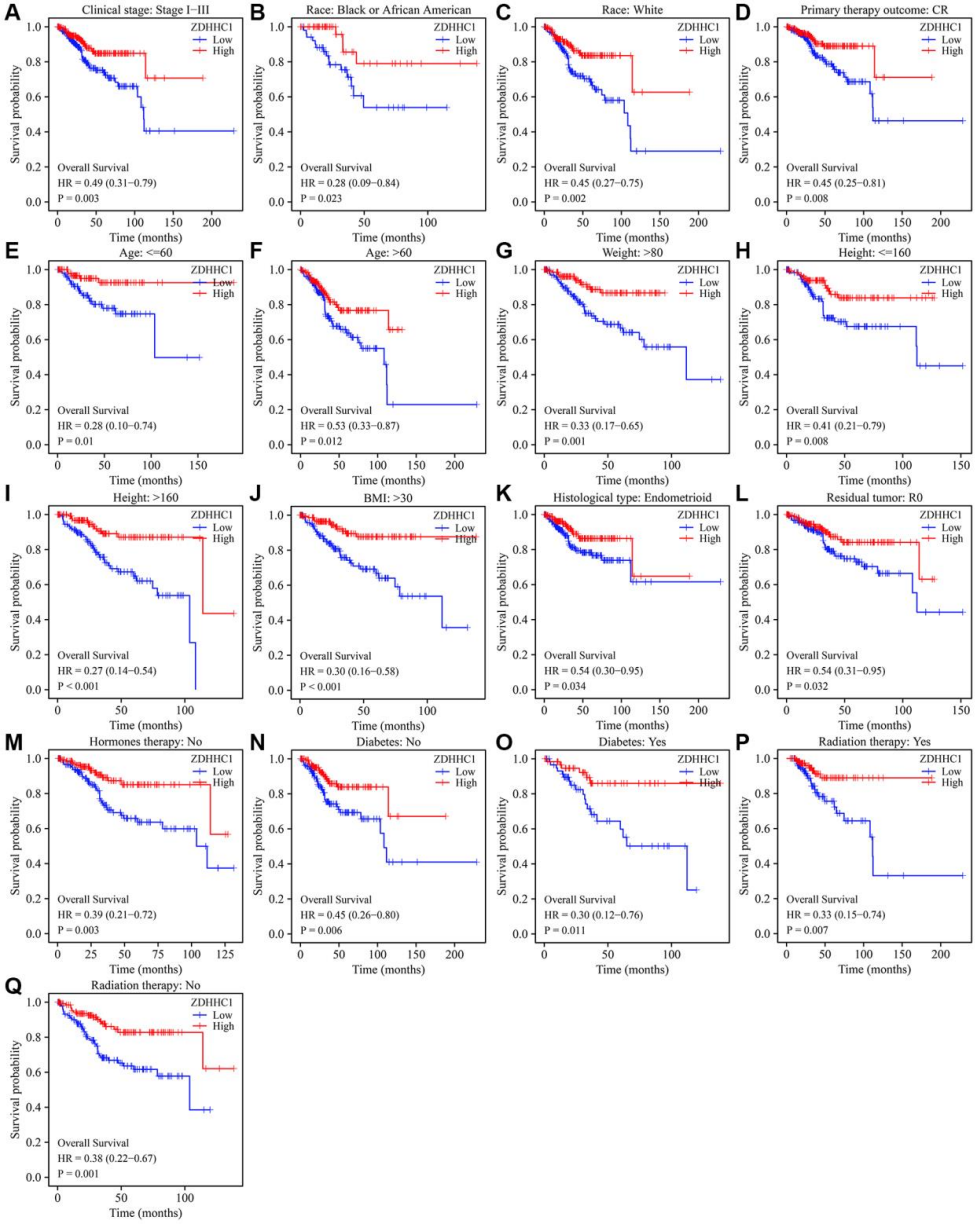
**ZDHHC1 expression is associated with poor survival in subgroups of patients with UCEC.** (**A**) Tumor stage I–III. (**B**) Black or African American. (**C**) White. (**D**) CR. (**E**) Age ≤60. (**F**) Age >60. (**G**) Weight >80 kg. (**H**) Height ≤160 cm. (**I**) Height >160 cm. (**J**) BMI >30. (**K**) Histological type of endometrioid. (**L**) R0. (**M**) Without hormone therapy. (**N**–**O**) With/without diabetes. (**P**, **Q**) With/without radiation therapy. Abbreviations: UCEC: uterine corpus endometrial carcinoma; ZDHHC1: zinc finger DHHC-type containing 1; CR: complete response; BMI: body mass index.

**Figure 5:**
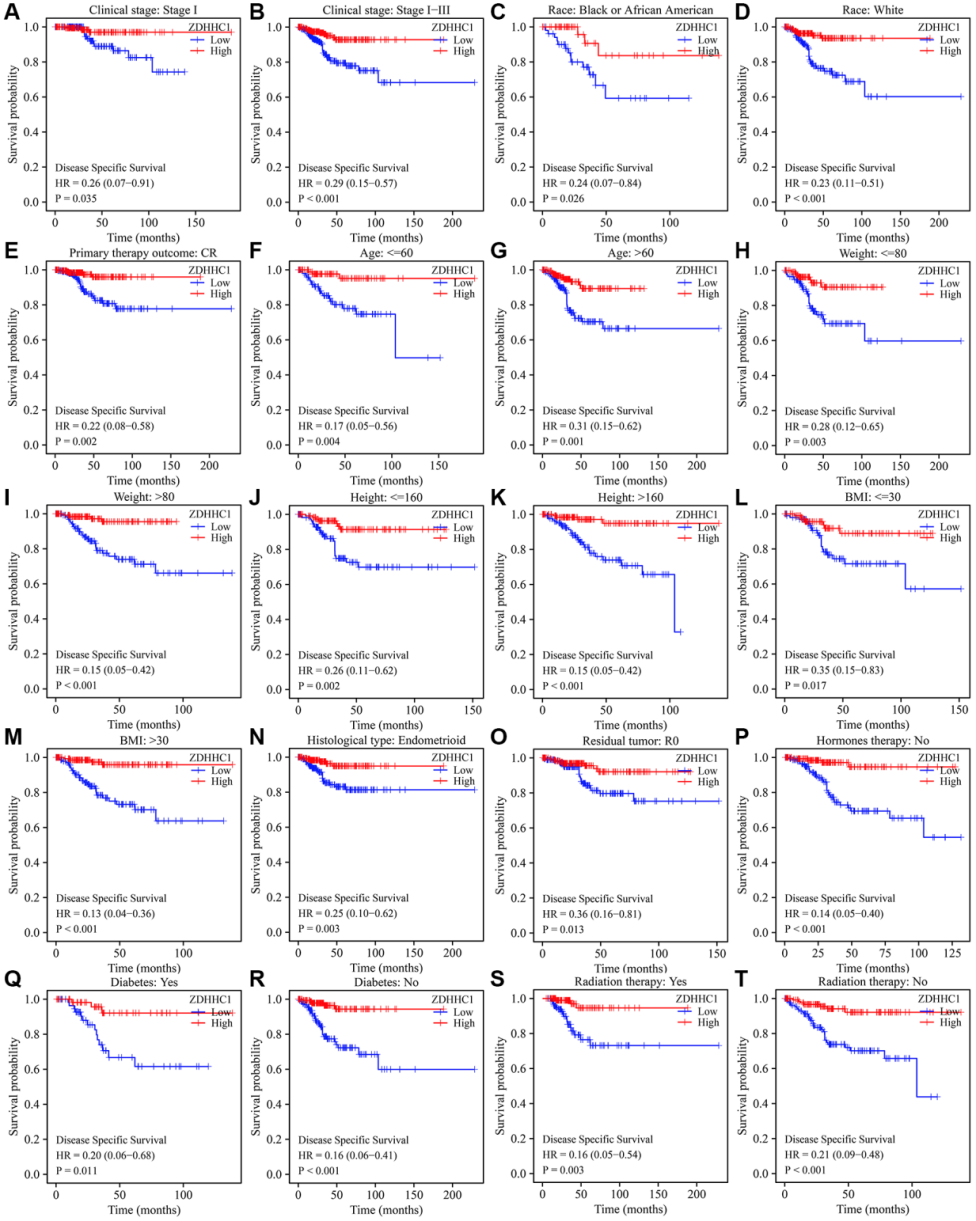
**ZDHHC1 expression is related to disease-specific survival in subgroups of patients with UCEC.** (**A**) Tumor stage I. (**B**) Tumor stage I–III. (**C**) Black or African American. (**D**) White. (**E**) CR. (**F**) Age ≤60. (**G**) Age >60. (**H**) Weight ≤80 kg. (**I**) Weight >80 kg. (**J**) Height ≤160 cm. (**K**) Height >160 cm. (**L**) BMI ≤30. (**M**) BMI >30. (**N**) Histological type of endometrioid. (**O**) R0. (**P**) Without hormone therapy. (**Q**, **R**) With/without diabetes. (**S**, **T**) With/without radiation therapy. Abbreviations: UCEC: uterine corpus endometrial carcinoma; ZDHHC1: zinc finger DHHC-type containing 1; CR: complete response; BMI: body mass index; R0: residual tumor.

A Kaplan-Meier survival analysis revealed that decreased ZDHHC1 expression significantly correlated with shorter OS among patients with the following features: clinical stage I–III, complete response (CR), residual tumor (R0), histological type of endometrioid, Black or African American, white, age ≤60, age >60, weight >80, height ≤160 cm, height >160 cm, BMI >30, with or without diabetes, no hormone therapy, and with or without radiation therapy (Figure 4). Furthermore, decreased ZDHHC1 expression significantly correlated with shorter DSS among patients with the following characteristics: clinical stage I or I–III, CR, R0, histological type of endometrioid, Black or African American, white, age ≤60, age >60, weight ≤80, weight >80, height ≤160 cm, height >160 cm, BMI ≤30, BMI >30, with or without diabetes, no hormone therapy, and with or without radiation therapy (Figure 5). Reduced ZDHHC1 expression also correlated with shorter PFI among patients with the following properties: clinical stage I or I–III, CR, R0, histological type of endometrioid, Black or African American, white, age ≤60, age >60, weight >80, height ≤160 cm, height >160 cm, BMI >30, with or without diabetes, no hormone therapy, and with or without radiation therapy (Supplementary Figure 1).

### Construction of nomograms for clinical outcome evaluation

**Figure 6:**
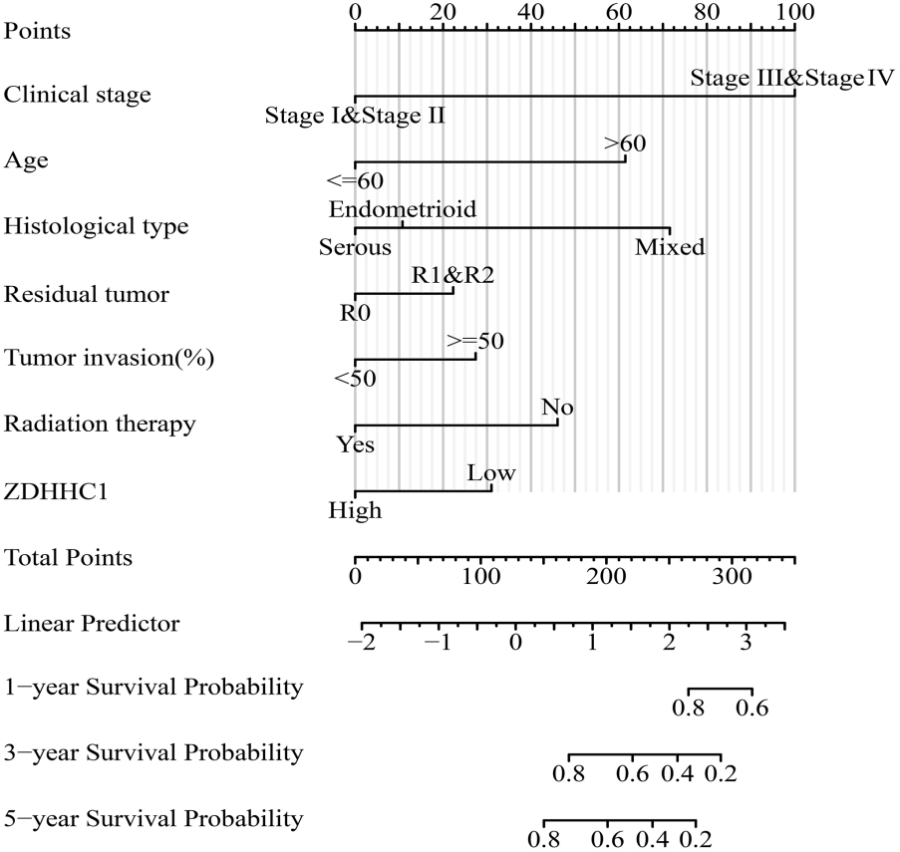
**Prognostic nomogram based on ZDHHC1 expression and OS of patients with UCEC.** Abbreviations: UCEC: uterine corpus endometrial carcinoma; ZDHHC1: zinc finger DHHC-type containing 1; OS: overall survival.

A Cox regression analysis indicated that several factors, including clinical stage, age, histological subtype, residual tumor, tumor invasion, and ZDHHC1 expression, were associated with a reduced OS among patients with UCEC (Table 1). Similarly, clinical stage, histological subtype, residual tumor, tumor invasion, and ZDHHC1 expression were significantly associated with a shorter DSS and PFI in the patients (Tables 2 and 3). We also constructed nomograms based on ZDHHC1 expression and prognostic indicators of OS, DSS, and PFI using Cox regression results and provided a comprehensive assessment of patient outcomes (Figure 6 and Supplementary Figure 2).

**Table 1:** OS-related factors in UCEC.

**Characteristics**	**Total (*N*)**	**HR (95% CI)**	***P*-value**
Clinical stage	551		
Stage I	341	Reference	
Stage II	51	1.751 (0.840–3.653)	0.135
Stage III	130	3.078 (1.907–4.968)	<0.001
Stage IV	29	8.065 (4.488–14.495)	<0.001
Age	549		
≤60	206	Reference	
>60	343	1.847 (1.160–2.940)	0.010
Weight	527		
≤80	242	Reference	
>80	285	1.060 (0.699–1.607)	0.784
Height	522		
≤160	246	Reference	
>160	276	1.153 (0.758–1.753)	0.507
BMI	518		
≤30	211	Reference	
>30	307	0.967 (0.636–1.470)	0.876
Histological type	551		
Endometrioid	409	Reference	
Mixed	24	2.421 (1.036–5.655)	0.041
Serous	118	2.667 (1.739–4.088)	<0.001
Residual tumor	412		
R0	374	Reference	
R1	22	1.578 (0.630–3.955)	0.331
R2	16	5.527 (2.879–10.612)	<0.001
Tumor invasion	473		
<50	259	Reference	
≥50	214	2.813 (1.744–4.535)	<0.001
Hormones therapy	344		
No	297	Reference	
Yes	47	0.801 (0.380–1.689)	0.560
Radiation therapy	527		
No	279	Reference	
Yes	248	0.594 (0.385–0.915)	0.018
ZDHHC1	551		
Low	275	Reference	
High	276	0.405 (0.260–0.630)	<0.001

Abbreviations: UCEC: uterine corpus endometrial carcinoma; OS: overall survival.

**Table 2:** DSS-related factors in UCEC.

**Characteristics**	**Total (*N*)**	**HR (95% CI)**	***P*-value**
Clinical stage	549		
Stage I	340	Reference	
Stage II	50	1.785 (0.592–5.382)	0.304
Stage III	130	5.935 (3.160–11.145)	<0.001
Stage IV	29	16.815 (8.274–34.173)	<0.001
Age	547		
≤60	206	Reference	
>60	341	1.215 (0.724–2.042)	0.461
Weight	525		
≤80	241	Reference	
>80	284	0.912 (0.551–1.510)	0.721
Height	520		
≤160	244	Reference	
>160	276	0.886 (0.533–1.472)	0.640
BMI	516		
≤30	210	Reference	
>30	306	0.948 (0.569–1.581)	0.839
Histological type	549		
Endometrioid	407	Reference	
Mixed	24	3.981 (1.651–9.599)	0.002
Serous	118	3.493 (2.071–5.891)	<0.001
Residual tumor	412		
R0	374	Reference	
R1	22	2.705 (1.049–6.974)	0.040
R2	16	9.442 (4.728–18.856)	<0.001
Tumor invasion	473		
<50	259	Reference	
≥50	214	3.281 (1.799–5.983)	<0.001
Hormones therapy	344		
No	297	Reference	
Yes	47	0.786 (0.307–2.011)	0.616
Radiation therapy	525		
No	277	Reference	
Yes	248	0.599 (0.351–1.021)	0.060
ZDHHC1	549		
Low	274	Reference	
High	275	0.224 (0.119–0.420)	<0.001

Abbreviations: UCEC: uterine corpus endometrial carcinoma; DSS: disease-specific survival.

**Table 3:** PFI-related factors in UCEC.

**Characteristics**	**Total (*N*)**	**HR (95% CI)**	***P*-value**
Clinical stage	551		
Stage I	341	Reference	
Stage II	51	1.016 (0.502–2.058)	0.965
Stage III	130	2.581 (1.740–3.827)	<0.001
Stage IV	29	6.832 (4.081–11.437)	<0.001
Age	549		
≤60	206	Reference	
>60	343	1.353 (0.934–1.961)	0.110
Weight	527		
≤80	242	Reference	
>80	285	1.035 (0.727–1.473)	0.848
Height	522		
≤160	246	Reference	
>160	276	1.016 (0.713–1.450)	0.929
BMI	518		
≤30	211	Reference	
>30	307	1.046 (0.730–1.500)	0.805
Histological type	551		
Endometrioid	409	Reference	
Mixed	24	2.035 (0.981–4.221)	0.056
Serous	118	2.123 (1.464–3.078)	<0.001
Residual tumor	412		
R0	374	Reference	
R1	22	1.396 (0.607–3.213)	0.433
R2	16	5.209 (2.821–9.621)	<0.001
Tumor invasion	473		
<50	259	Reference	
≥50	214	1.885 (1.289–2.756)	0.001
Hormones therapy	344		
No	297	Reference	
Yes	47	1.250 (0.700–2.232)	0.450
Radiation therapy	527		
No	279	Reference	
Yes	248	1.095 (0.771–1.556)	0.613
ZDHHC1	551		
Low	275	Reference	
High	276	0.452 (0.313–0.654)	<0.001

Abbreviations: UCEC: uterine corpus endometrial carcinoma; PFI: progression-free interval.

### Functions, mechanisms, and protein-protein interaction (PPI) networks associated with ZDHHC1

**Figure 7:**
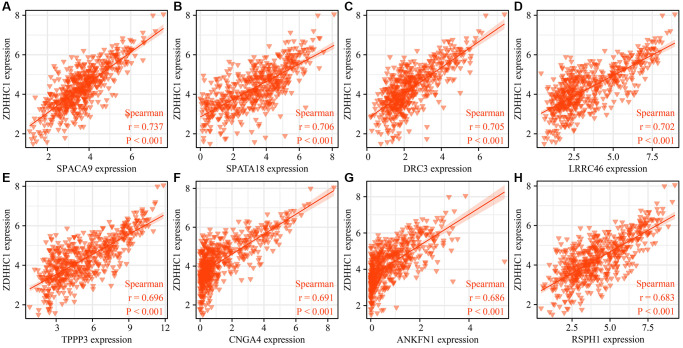
**Visualization of ZDHHC1 co-expressed genes.** (**A**) SPACA9. (**B**) SPATA18. (**C**) DRC3. (**D**) LRRC46. (**E**) TPPP3. (**F**) CNGA4. (**G**) ANKFN1. (**H**) RSPH1. Abbreviations: ZDHHC1: zinc finger DHHC-type containing 1; SPACA9: sperm acrosome associated 9; SPATA18: spermatogenesis associated 18; DRC3: dynein regulatory complex subunit 3; LRRC46: leucine rich repeat containing 46; TPPP3: tubulin polymerization promoting protein family member 3; CNGA4: cyclic nucleotide gated channel subunit alpha 4; ANKFN1: ankyrin repeat and fibronectin type III domain containing 1; RSPH1: radial spoke head component 1.

**Figure 8:**
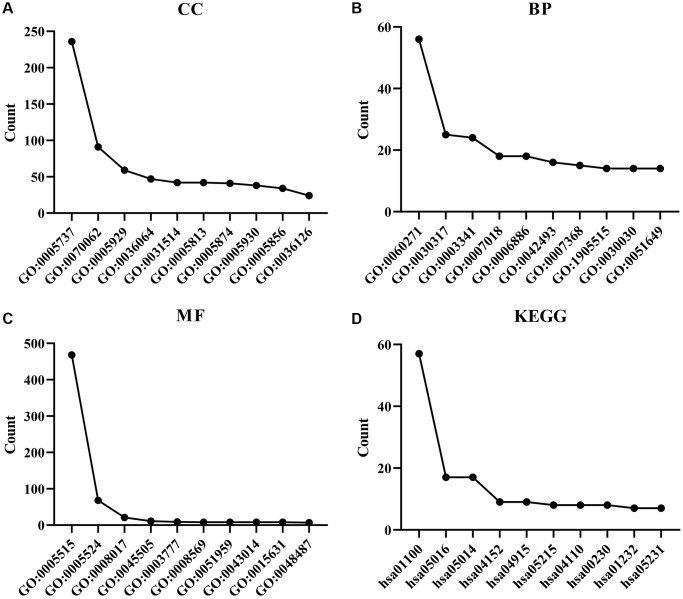
**Functions and molecular pathways of ZDHHC1 co-expressed genes.** (**A**–**C**) GO analysis. (**D**) Molecular pathways uncovered using KEGG analysis. Abbreviations: ZDHHC1: zinc finger DHHC-type containing 1; BP: biological process; KEGG: Kyoto Encyclopedia of Genes and Genomes; MF: molecular function; CC: cell component; GO: Gene Ontology.

**Figure 9:**
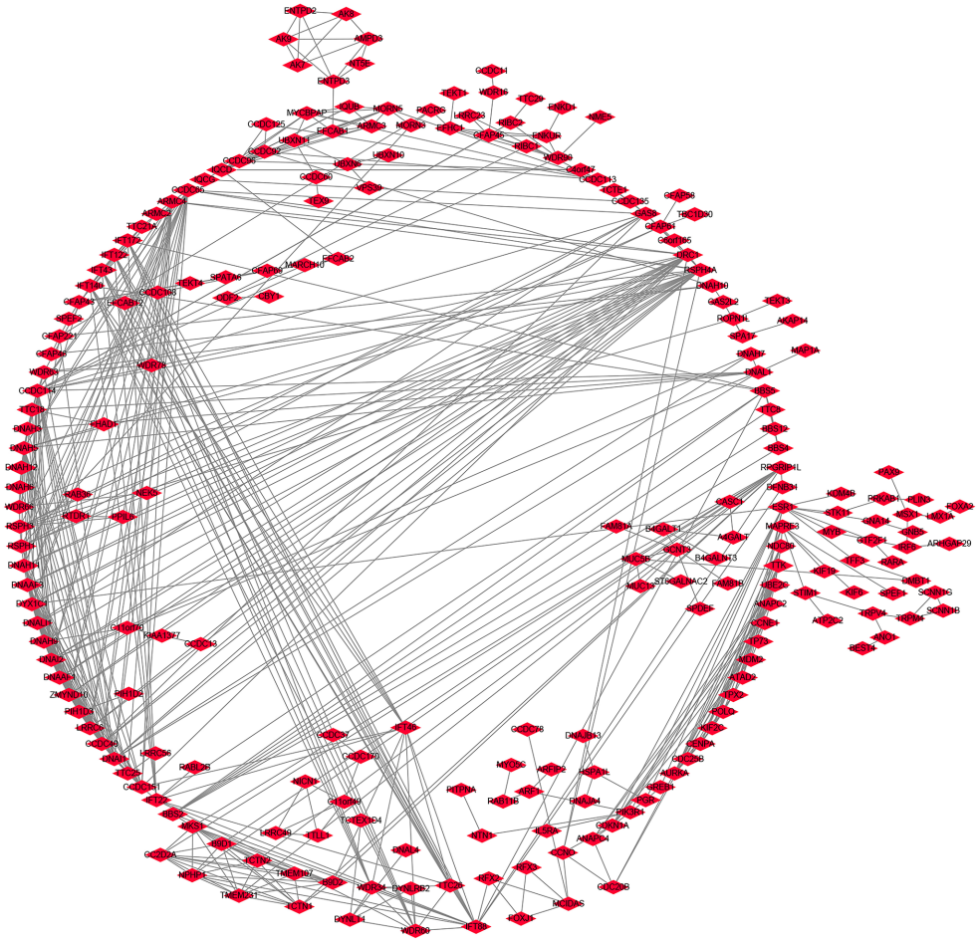
A protein network of ZDHHC1 co-expressed genes.

A total of 825 genes were co-expressed with the ZDHHC1 gene as revealed by a correlation analysis (Figure 7 and Supplementary Table 1). These genes were involved in intracellular protein transport, negative regulation of protein binding, and regulation of the mitotic cell cycle, and other functions (Figure 8A–8C and Supplementary Table 2). In addition, they were enriched in several signaling pathways, such as AMP-activated protein kinase (AMPK), p53, and cell cycle signaling pathways (Figure 8D and Supplementary Table 2). Next, GSEA was performed and showed that ZDHHC1 expression was linked to drug metabolism, PI3K/AKT signaling pathway, spliceosome, Toll-like receptor signaling pathway, cell adhesion molecules, cell cycle, and DNA replication (Table 4). Finally, a protein network of the genes co-expressed with ZDHHC1 was constructed and is depicted in Figure 9.

**Table 4:** Pathways associated with ZDHHC1 expression.

**Type**	**Description**	**Count**	***P*-value**
hsa00730	Thiamine metabolism	15	0.011616656
hsa00071	Fatty acid degradation	43	0.005091566
hsa00350	Tyrosine metabolism	36	0.01302842
hsa00982	Drug metabolism-cytochrome P450	59	0.014338491
hsa00010	Glycolysis/Gluconeogenesis	60	0.027693709
hsa04740	Olfactory transduction	96	0.01097699
hsa05150	Staphylococcus aureus infection	85	0.015488993
hsa04915	Estrogen signaling pathway	135	0.031934089
hsa05016	Huntington disease	287	0.014821499
hsa04151	PI3K-Akt signaling pathway	330	0.041800643
hsa05206	MicroRNAs in cancer	168	0.041426928
hsa03040	Spliceosome	136	0.030989273
hsa04725	Cholinergic synapse	111	0.020831228
hsa05222	Small cell lung cancer	92	0.038653367
hsa04972	Pancreatic secretion	95	0.025397632
hsa04726	Serotonergic synapse	110	0.005265464
hsa04620	Toll-like receptor signaling pathway	87	0.022294811
hsa05160	Hepatitis C	138	0.002532147
hsa04514	Cell adhesion molecules	141	0.001247981
hsa04110	Cell cycle	125	0.003388987
hsa04721	Synaptic vesicle cycle	76	0.024063466
hsa03008	Ribosome biogenesis in eukaryotes	79	0.006483165
hsa04512	ECM-receptor interaction	86	0.005722247
hsa04923	Regulation of lipolysis in adipocytes	52	0.049731183
hsa04080	Neuroactive ligand-receptor interaction	307	3.95E-08
hsa04911	Insulin secretion	82	0.004534427
hsa05032	Morphine addiction	85	0.002843451
hsa04976	Bile secretion	78	0.003742149
hsa04979	Cholesterol metabolism	49	0.015978484
hsa04978	Mineral absorption	58	0.016923453
hsa04260	Cardiac muscle contraction	82	0.001452873
hsa04930	Type II diabetes mellitus	45	0.019333534
hsa05143	African trypanosomiasis	34	0.042699725
hsa04727	GABAergic synapse	84	0.000452689
hsa05340	Primary immunodeficiency	37	0.02356343
hsa04742	Taste transduction	67	0.002059442
hsa01040	Biosynthesis of unsaturated fatty acids	27	0.043417367
hsa00062	Fatty acid elongation	27	0.042016807
hsa00052	Galactose metabolism	29	0.040248415
hsa05033	Nicotine addiction	37	0.016911238
hsa01523	Antifolate resistance	31	0.021178684
hsa03030	DNA replication	36	0.007474553
hsa04973	Carbohydrate digestion and absorption	40	0.005302208
hsa04974	Protein digestion and absorption	99	1.68E-06
hsa00220	Arginine biosynthesis	22	0.007180534
hsa04964	Proximal tubule bicarbonate reclamation	22	0.002591659

### Increasing ZDHHC1 expression inhibits growth and metastasis of UCEC cells

**Figure 10:**
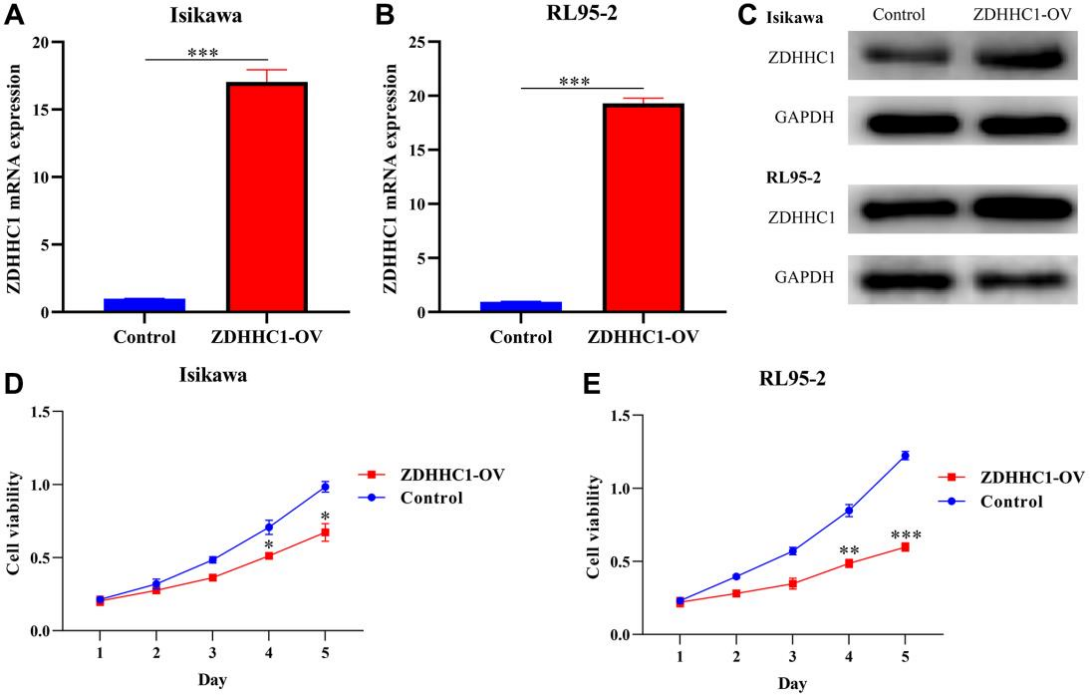
**ZDHHC1 overexpression prevents UCEC cell proliferation.** (**A**–**C**) ZDHHC1 is overexpressed in UCEC cells. (**D**, **E**) Cell proliferation assessed with CCK-8 assay. Abbreviations: UCEC: uterine corpus endometrial carcinoma; ZDHHC1: zinc finger DHHC-type containing 1; CCK-8: Cell counting kit-8.

**Figure 11:**
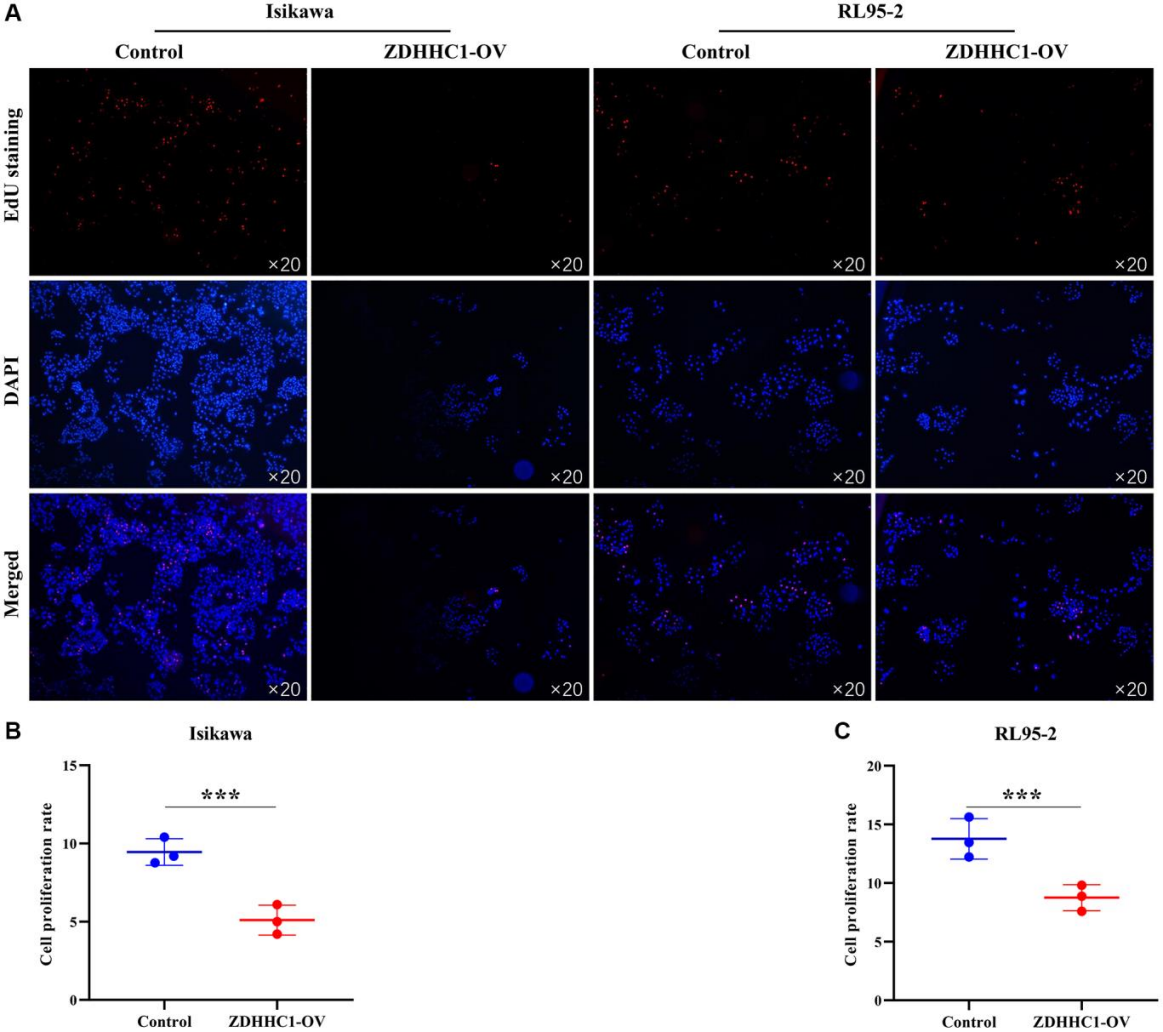
**ZDHHC1 overexpression prevents UCEC cell proliferation.** (**A**) Cell proliferation using EdU assay; (**B**, **C**) The ability of cell proliferation was analyzed statistically. Abbreviations: UCEC: uterine corpus endometrial carcinoma; ZDHHC1: zinc finger DHHC-type containing 1.

**Figure 12:**
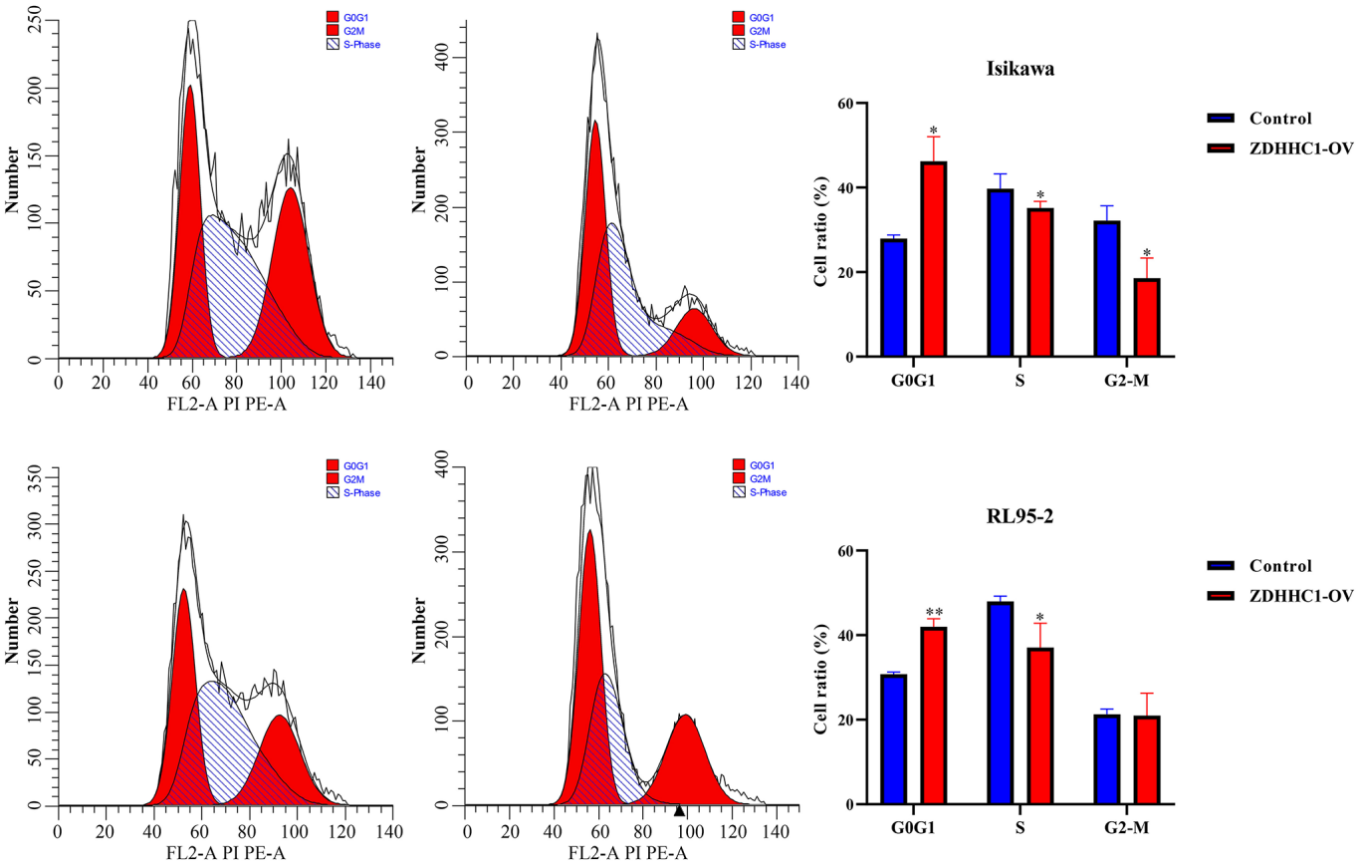
**ZDHHC1 overexpression suppresses the cell cycle in UCEC.** Abbreviations: UCEC: uterine corpus endometrial carcinoma; ZDHHC1: zinc finger DHHC-type containing 1.

**Figure 13:**
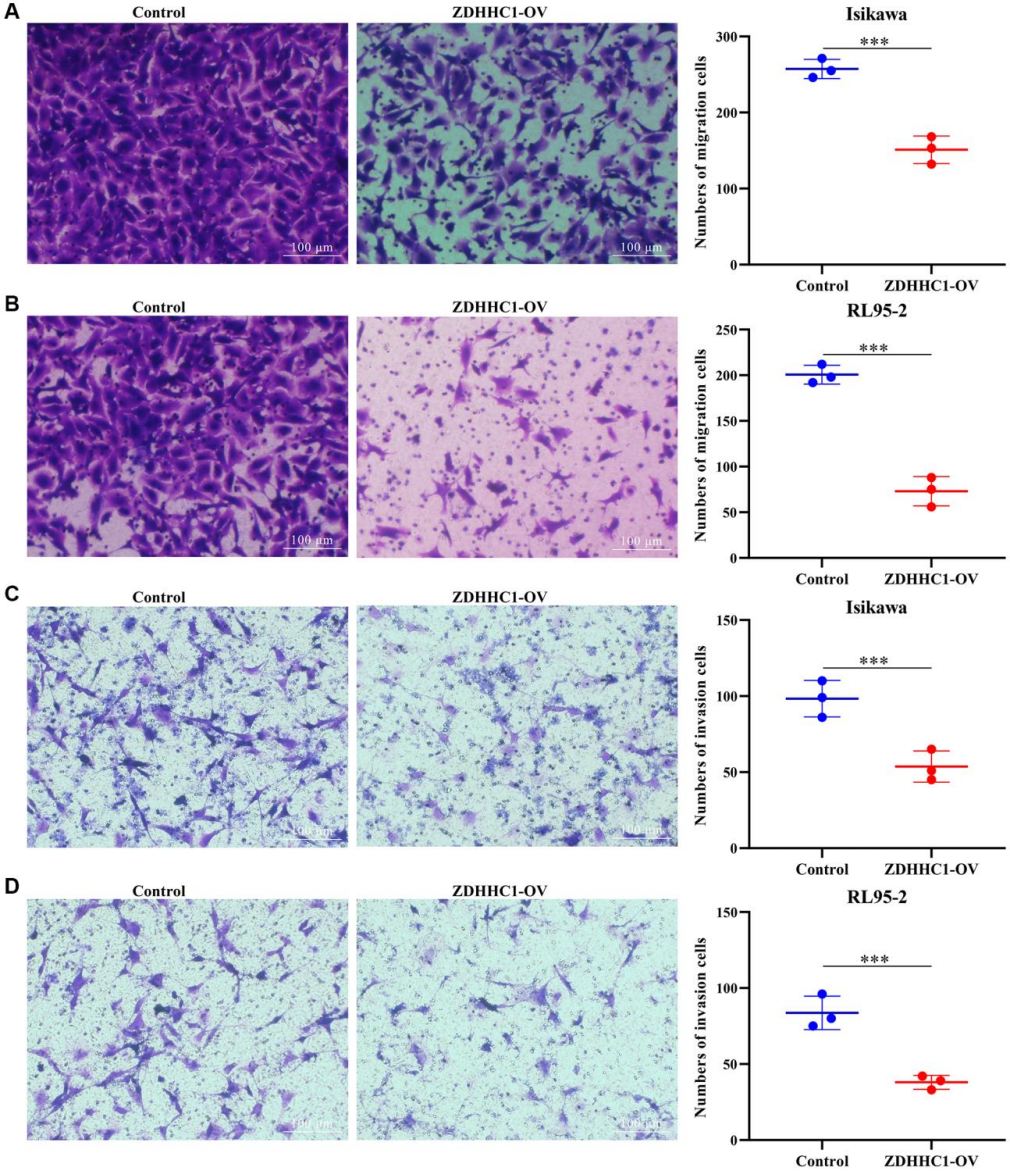
**ZDHHC1 overexpression hinders migration and invasion of UCEC cells.** (**A**, **B**) Cell migration; (**C**, **D**) Cell invasion. Abbreviations: UCEC: uterine corpus endometrial carcinoma; ZDHHC1: zinc finger DHHC-type containing 1.

Human endometrial and adenosquamous carcinoma cells were transfected with a plasmid expressing the ZDHHC1 gene or control plasmid to generate ZDHHC1 overexpression or control lines. The lines were verified with RT-PCR and Western blotting (Figure 10A–10C), and the effect of ZDHHC1 overexpression on cell proliferation and migration was explored. A CCK-8 assay showed that ZDHHC1 overexpression significantly inhibited cell proliferation in ZDHHC1 overexpression cells versus control cells, with a significant statistical difference in cell absorbance between them at 72 h and 96 h (Figure 10D, 10E). Moreover, EdU staining confirmed that ZDHHC1 overexpression significantly repressed cell proliferation in ZDHHC1 overexpression cells (Figure 11A–11C). In addition, flow cytometry showed that ZDHHC1 overexpression significantly inhibited cell cycle transition, and a Transwell assay demonstrated that above-normal ZDHHC1 expression significantly hindered cell invasion and migration (Figures 12 and 13).

### ZDHHC1 expression is associated with immune microenvironment of UCEC

**Figure 14:**
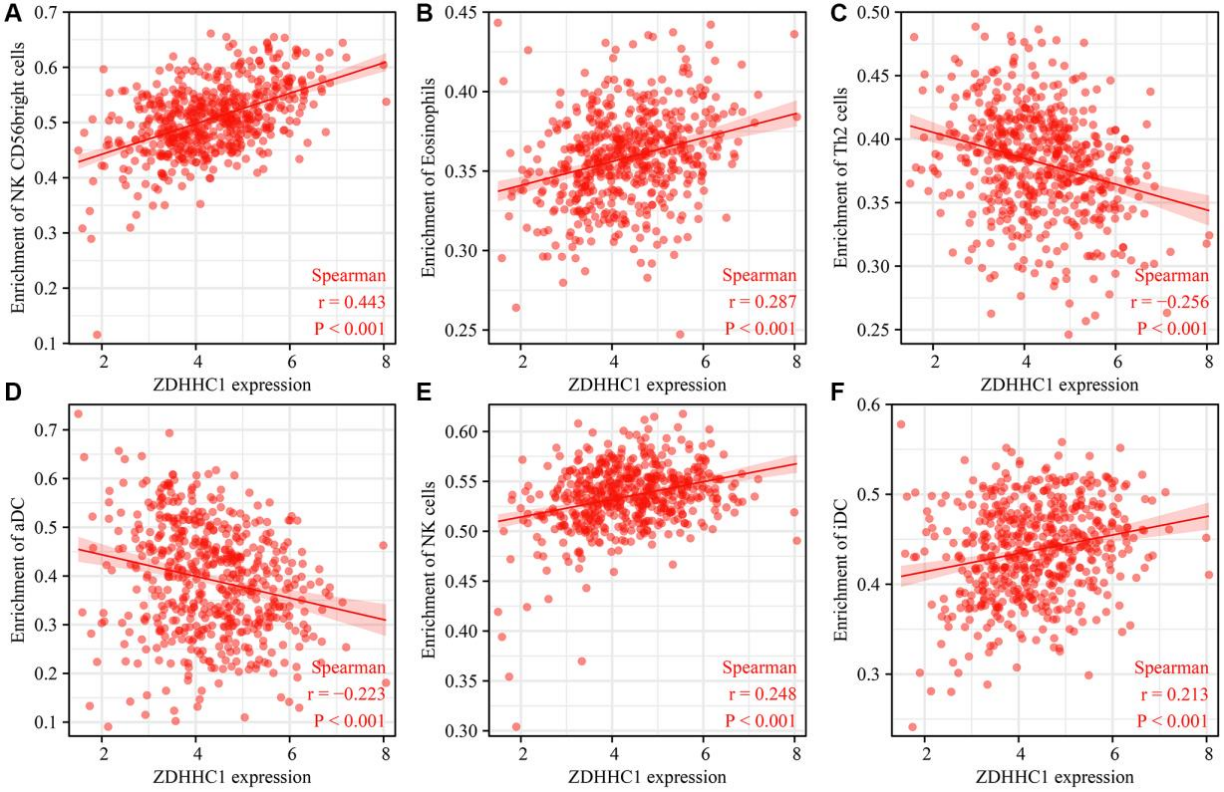
**Correlation between ZDHHC1 overexpression and tumor-infiltrating immune cells.** (**A**) NK CD56bright cells; (**B**) Eosinophils; (**C**) Th2 cells; (**D**) aDC; (**E**) NK cells; (**F**) iDC.

A significant correlation was found between ZDHHC1 expression and the levels of various tumor-infiltrating immune cells, including CD56 bright NK cells (r = 0.443), eosinophils (r = 0.287), T helper 2 (Th2) cells (r = −0.256), NK cells (r = 0.248), activated dendritic cells (aDCs) (r = −0.223), immature DCs (iDCs) (r = 0.213), macrophages (r = −0.211), Th17 cells (r = 0.206), Th1 cells (r = −0.176), T helper cells (r = −0.162), mast cells (r = 0.159), gamma delta T (gdT) cells (r = −0.141), B cells (r = −0.137), neutrophils (r = 0.126), and DCs (r = −0.110) (Figure 14 and Table 5). Expression levels of these tumor-infiltrating immune cells in high- and low-ZDHHC1 expression groups are illustrated in Supplementary Figure 3. In addition, a correlation analysis showed ZDHHC1 expression significantly correlated that of various immune cell markers, including IL10, CSF1R, CD163, CD68, ITGAM, PTGS2, HLA-DPB1, CCR7, CD19, CD3E, MS4A4A, CD3D, CD86, CD1C, STAT6, STAT1, STAT5B, STAT3, LAG3, HLA-DRA, STAT5A, NRP1, IFNG, HLA-DQB1, GZMB, IL13, HLA-DPA1, and GATA3 (Table 6). An analysis of the TIMER database also revealed correlations between ZDHHC1 expression and that of many immune cell markers (Supplementary Figure 4 and Table 6).

**Table 5:** Correlation between ZDHHC1 overexpression and tumor-infiltrating immune cells.

**Gene**	**Immune cells**	**Correlation coefficient**	***P*-value**
ZDHHC1	aDC	−0.223	<0.001
ZDHHC1	B cells	−0.137	0.001
ZDHHC1	CD8 T cells	−0.014	0.746
ZDHHC1	Cytotoxic cells	0.019	0.651
ZDHHC1	DC	−0.110	0.009
ZDHHC1	Eosinophils	0.287	<0.001
ZDHHC1	iDC	0.213	<0.001
ZDHHC1	Macrophages	−0.211	<0.001
ZDHHC1	Mast cells	0.159	<0.001
ZDHHC1	Neutrophils	0.126	0.003
ZDHHC1	NK CD56bright cells	0.443	<0.001
ZDHHC1	NK CD56dim cells	−0.020	0.636
ZDHHC1	NK cells	0.248	<0.001
ZDHHC1	pDC	0.037	0.390
ZDHHC1	T cells	0.037	0.385
ZDHHC1	T helper cells	−0.162	<0.001
ZDHHC1	Tcm	−0.038	0.368
ZDHHC1	Tem	−0.037	0.383
ZDHHC1	TFH	−0.026	0.541
ZDHHC1	Tgd	−0.141	<0.001
ZDHHC1	Th1 cells	−0.176	<0.001
ZDHHC1	Th17 cells	0.206	<0.001
ZDHHC1	Th2 cells	−0.256	<0.001
ZDHHC1	TReg	−0.018	0.678

**Table 6:** Correlation between ZDHHC1 overexpression and immune cell markers.

**Markers**	**TCGA database**		**TIMER database**	
	**Coefficient**	***P*-value**	**Coefficient**	***P*-value**
CD1C	0.363	<0.001	0.375932662	9.75E-20
STAT6	0.336	<0.001	0.331458861	2.55E-15
STAT1	−0.326	<0.001	−0.309230354	2.03E-13
STAT5B	0.272	<0.001	0.290438137	5.90E-12
STAT3	0.216	<0.001	0.256473988	1.24E-09
LAG3	−0.199	<0.001	−0.233269631	3.95E-08
IL10	−0.196	<0.001	−0.174226322	4.33E-05
CSF1R	0.182	<0.001	0.185079215	1.42E-05
CD163	−0.178	<0.001	−0.164992292	0.000109063
CD68	−0.173	<0.001	−0.026376888	0.538789204
ITGAM	0.170	<0.001	0.188152683	9.78E-06
HLA-DRA	0.168	<0.001	0.132361024	0.001957941
PTGS2	0.154	<0.001	0.185521079	1.31E-05
HLA-DPB1	0.151	<0.001	0.150674804	0.000421968
STAT5A	0.149	<0.001	0.177427868	3.10E-05
NRP1	0.141	<0.001	0.157912625	0.000214505
IFNG	−0.137	0.001	−0.117432641	0.006056613
CCR7	0.127	0.003	0.143874946	0.000763999
HLA-DQB1	0.125	0.003	0.085443712	0.046201048
GZMB	−0.123	0.004	−0.14492396	0.000690189
IL13	0.119	0.005	0.052780384	0.218622599
CD19	0.117	0.006	0.100695275	0.018706107
HLA-DPA1	0.115	0.007	0.084012042	0.049989052
CD3E	0.113	0.008	0.109130131	0.010820403
MS4A4A	−0.107	0.012	−0.100012157	0.01956088
CD3D	0.099	0.020	0.068933029	0.107950112
GATA3	−0.093	0.030	−0.050786333	0.236547425
CD86	−0.092	0.031	−0.0938364	0.028492476

### ZDHHC1 expression is associated with RNA modifications

**Figure 15:**
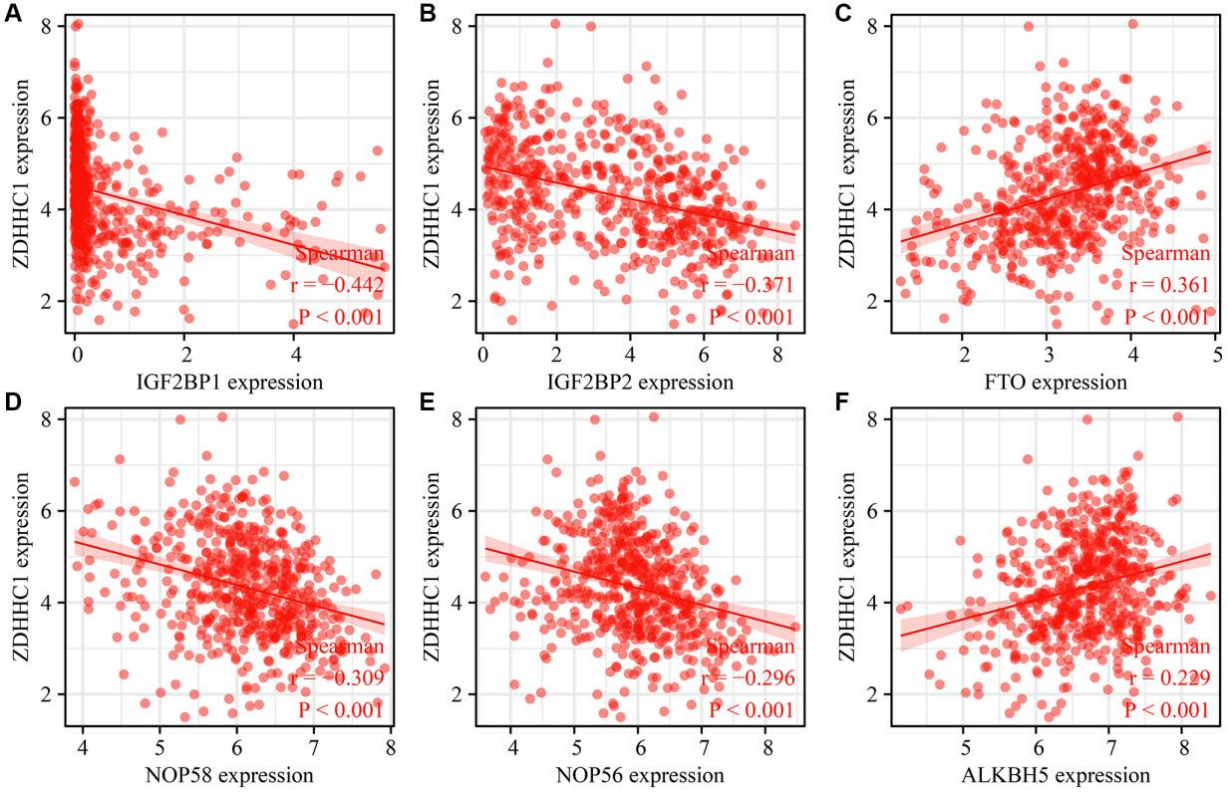
**Correlation between ZDHHC1 overexpression and RNA modification genes.** (**A**) IGF2BP1; (**B**) IGF2BP2; (**C**) FTO; (**D**) NOP56; (**E**) NOP58; (**F**) ALKBH5. Abbreviations: ZDHHC1, zinc finger DHHC-type containing 1; IGF2BP1/2, insulin like growth factor 2 mRNA binding protein 1/2; FTO, FTO alpha-ketoglutarate dependent dioxygenase; NOP56/58, NOP56/58 ribonucleoprotein; ALKBH5, ALKB homolog 5.

RNA modification genes were extracted from the RM2Target database, and ZDHHC1 expression significantly correlated with the expression levels of several RNA modification genes: IGF2BP1 (*P* < 0.001), IGF2BP2 (*P* < 0.001), FTO (*P* < 0.001), NOP58 (*P* < 0.001), NOP56 (*P* < 0.001), ALKBH5 (*P* < 0.001), DKC1 (*P* < 0.001), LRPPRC (*P* < 0.001), METTL1 (*P* < 0.001), METTL5 (*P* < 0.001), YBX1 (*P* < 0.001), ADARB1 (*P* < 0.001), YTHDF1 (*P* < 0.001), ELAVL1 (*P* < 0.001), YTHDC2 (*P* < 0.001), WTAP (*P* < 0.001), ALYREF (*P* = 0.001), FBL (*P* = 0.006), ZCCHC4 (*P* = 0.006), YTHDF3 (*P* = 0.009), and PCIF1 (*P* = 0.025) (Figure 15 and Table 7).

**Table 7:** Correlation between ZDHHC1 overexpression and RNA modifications.

**Genes of RNA modification**	**Coefficient**	***P*-value**
IGF2BP1	−0.442	<0.001
IGF2BP2	−0.371	<0.001
FTO	0.361	<0.001
NOP58	−0.309	<0.001
NOP56	−0.296	<0.001
ALKBH5	0.229	<0.001
DKC1	−0.218	<0.001
LRPPRC	−0.212	<0.001
METTL1	−0.193	<0.001
METTL5	−0.193	<0.001
YBX1	−0.192	<0.001
ADARB1	−0.173	<0.001
YTHDF1	−0.165	<0.001
ELAVL1	0.156	<0.001
YTHDC2	0.149	<0.001
WTAP	−0.142	<0.001
ALYREF	−0.137	0.001
FBL	−0.117	0.006
ZCCHC4	0.117	0.006
YTHDF3	−0.110	0.009
PCIF1	0.095	0.025
PUS7	−0.074	0.082
ALKBH1	−0.064	0.135
METTL3	0.064	0.132
HNRNPA2B1	−0.056	0.192
HNRNPC	−0.052	0.221
RBMX	−0.050	0.239
METTL14	0.045	0.294
WDR4	0.044	0.302
ADAR	−0.038	0.377
ZC3H13	0.026	0.537
YTHDF2	−0.010	0.821
YTHDC1	0.000	0.991

## DISCUSSION

Early diagnosis is often associated with better cancer prognosis since middle- and advanced-stage patients with cancer tend to have poor outcomes. Abundant evidence suggests that inhibiting oncogenes or enhancing the expression of tumor suppressor genes improves the survival time of patients with cancer [13, 15, 17, 18]. For instance, Yan et al. [17] reported that overexpressing microsomal glutathione S-transferase 1 (MGST1), which is associated with histological type of tumor, hormone therapy, and tumor immune cell infiltration, was linked to better survival outcomes in patients with UCEC. The ZDHHC1 gene is also implicated in tumor progression [8, 9], with enhanced expression promoting cancer cell apoptosis, inhibiting cell cycle arrest and metastasis, and inducing oxidative and endoplasmic reticulum stress [8]. However, the relationship between ZDHHC1 expression and UCEC progression has not been extensively studied, demanding a genomic and *in vitro* assessment. This study found that ZDHHC1 expression was lower in UCEC tissues than in normal endometrial tissues and considerably downregulated in deceased patients and those with advanced age and advanced clinical stage, serous subtype, and high histological grade of UCEC. Decreased ZDHHC1 expression indicated a significant diagnostic value in UCEC and was significantly associated with shorter OS, DSS, and PFI of patients with UCEC. Moreover, reduced ZDHHC1 expression was a risk factor for poor prognosis among patients with UCEC.

Cell growth, cell metastasis, and regulation of several signaling pathways, such as the PI3K/AKT, cell cycle, and DNA replication pathways, are critical steps for cancer progression [19–23]. For instance, harakiri, BCL2 interacting protein (HRK) is downregulated in colorectal cancer tissues and cells, and its below-normal expression promotes apoptosis and inhibits the PI3K/AKT signaling pathway, reducing growth and migration of colorectal cancer cells [19]. Similarly, cyclin dependent kinase inhibitor 3 (CDKN3) is overexpressed in prostate cancer, and its inhibition enhances apoptosis and promotes G1 cell cycle arrest by reducing the expression of cell cycle and DNA replication proteins, inhibiting cancer cell growth and invasion [22]. We performed GSEA in this study to suggest the possible mechanisms of ZDHHC1-mediated UCEC progression and found that ZDHHC1 expression was closely associated with PI3K/AKT signaling, cell cycle, and DNA replication pathways. Moreover, overexpressing ZDHHC1 in human cell lines significantly inhibited cell proliferation, cell cycle transition, cell invasion, and migration. Hence, these findings agree with previous literature reports and suggest that ZDHHC1 is potentially involved in UCEC progression via the known mechanisms.

The occurrence and development of UCEC are associated with abnormal events in the tumor immune microenvironment [24, 25]. Rousset-Rouviere et al. [24] found that chemotherapy-resistant patients with UCEC with high microsatellite instability showed a robust response to programmed death 1 (PD-1) and programmed death ligand 1 (PDL-1) inhibitors, with high efficacy observed for pembrolizumab combined with the angiogenesis inhibitor lenvatinib. Our study analyzed the correlation between tumor-infiltrating immune cells and ZDHHC1 expression in UCEC tissues and showed that ZDHHC1 expression significantly correlated with levels of various tumor-infiltrating immune cells and immune cell markers. Another essential aspect of UCEC progression is RNA modifications since many RNA modification-associated genes exhibit robust predictive capability for UCEC prognosis and substantial influence on immune infiltration [26–29]. For example, high expression of insulin like growth factor 2 mRNA binding protein 1 (IGF2BP1) is associated with poor prognosis in patients with UCEC. Its expression promotes cell proliferation and the cell cycle through the m6A-dependent regulatory mechanism, stimulating tumor progression [26]. This study searched RNA modification genes from the RM2Target database that are related to ZDHHC1 expression and showed that various RNA modification genes, such as IGF2BP1, FTO, and METTL5, significantly correlated with ZDHHC1 expression in UCEC. These findings suggest that ZDHHC1 has the potential as a marker gene for predicting the poor prognosis of patients with UCEC.

Our comprehensive analysis uncovered involvement of ZDHHC1 in the mechanisms allowing UCEC progression, including crucial signaling pathways that promote cancer progression. Below-normal ZDHHC1 expression in UCEC strongly correlates with poor prognostic factors, while ZDHHC1 overexpression inhibits the cell growth, cycle transition, migration, and invasion of UCEC cells. Moreover, ZDHHC1 expression significantly correlates with cancer immune cells, cell markers, and RNA modifications, making ZDHHC1 a promising prognostic marker for UCEC. A limitation of this study is that ZDHHC1 involvement in regulating cell cycle progression, DNA replication, and PI3K-AKT signaling was not confirmed with Western blotting, and extensive clinical validation should be performed to confirm the prognostic value of ZDHHC1. Future studies should also consider collecting more tissue samples to explore the clinical importance of ZDHHC1. In conclusion, downregulated ZDHHC1 expression is associated with cancer cell growth, metastasis, poor prognosis, tumor immune infiltration, and RNA modifications in UCEC, emphasizing ZDHHC1 potential as a prognostic marker for the prognosis of patients with UCEC.

## Supplementary Materials

Supplementary Figures

Supplementary Tables
