# Supplementary material for: Overexpression of zinc finger DHHC-type containing 1 is associated with poor prognosis and cancer cell growth and metastasis in uterine corpus endometrial carcinoma
Source: Aging (Albany NY). 2024 Jun 6;16(11):9784–812. doi: 10.18632/aging.205899 (PMC11210219; doi:10.18632/aging.205899)
Supplement: Supplementary Figures [file aging-16-205899-s001.pdf]

SUPPLEMENTARY FIGURES

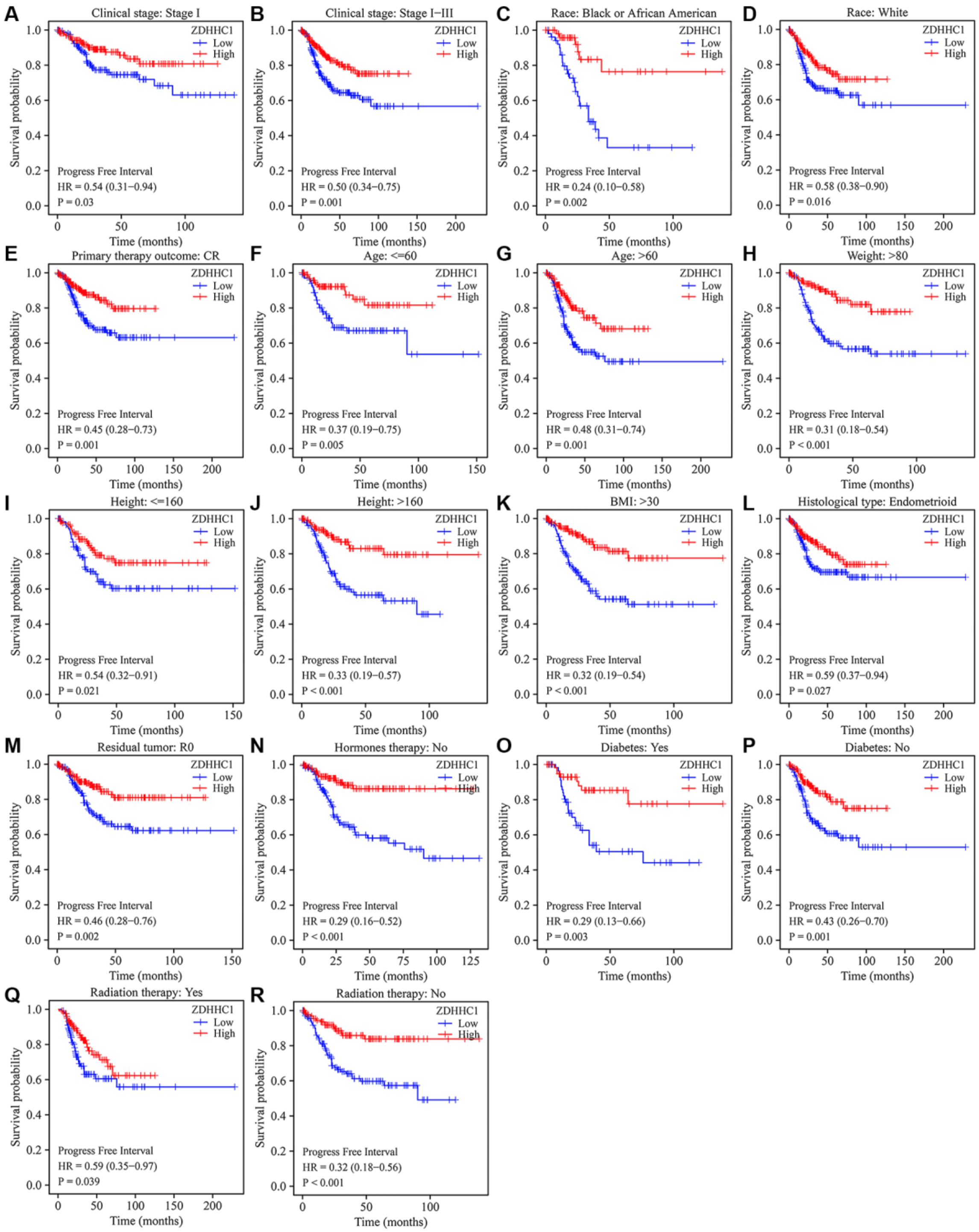

Supplementary Figure 1. ZDHHC1 expression is related to DSS in subgroups of patients with UCEC. (**A**) Tumor stage I. (**B**) Tumor stage I–III. (**C**) Black or African American. (**D**) White. (**E**) CR. (**F**) Age ≤60. (**G**) Age >60. (**H**) Weight >80 kg. (**I**) Height ≤160 cm.(**J**) Height >160 cm. (**K**) BMI >30. (**L**) Histological type of endometrioid. (**M**) R0. (**N**) Without hormone therapy. (**O**, **P**) With/without diabetes.(**Q**, **R**) With/without radiation therapy. Abbreviations: UCEC: uterine corpus endometrial carcinoma; DSS: disease-specific survival; CR: complete response; R0: residual tumor.

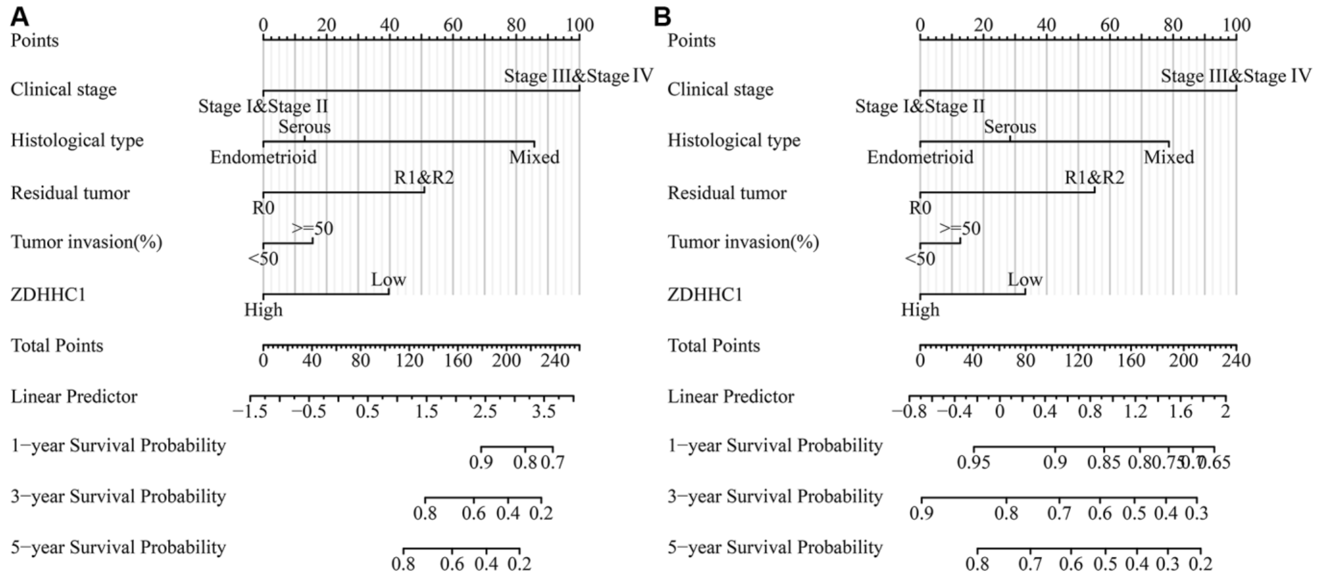

Supplementary Figure 2. Prognostic nomograms based on ZDHHC1 expression and DSS and PFI of UCEC patients. (**A**) DSS; (**B**) PFI.

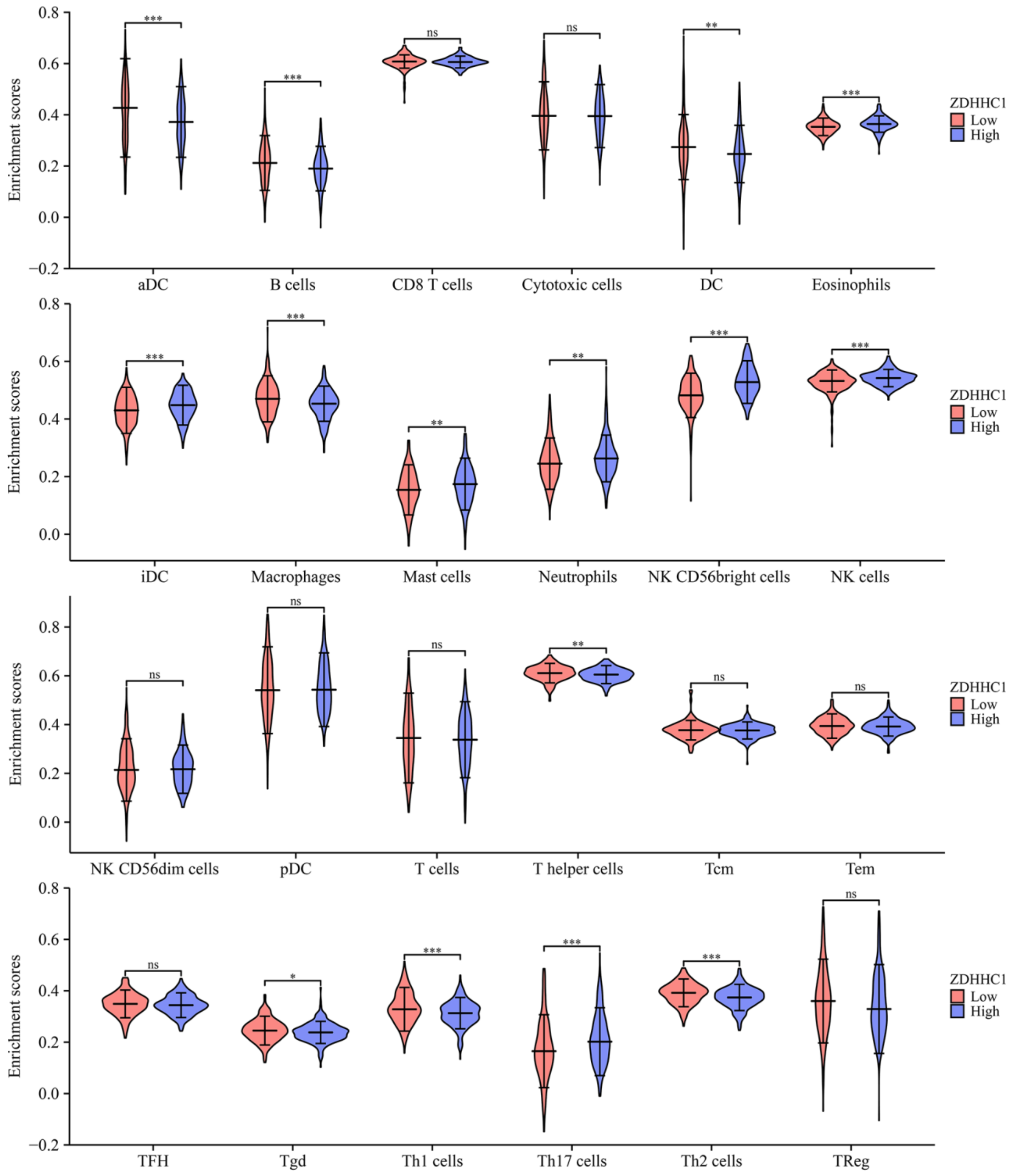

Supplementary Figure 3. Tumor-infiltrating immune cells in high- and low-ZDHHC1 groups.

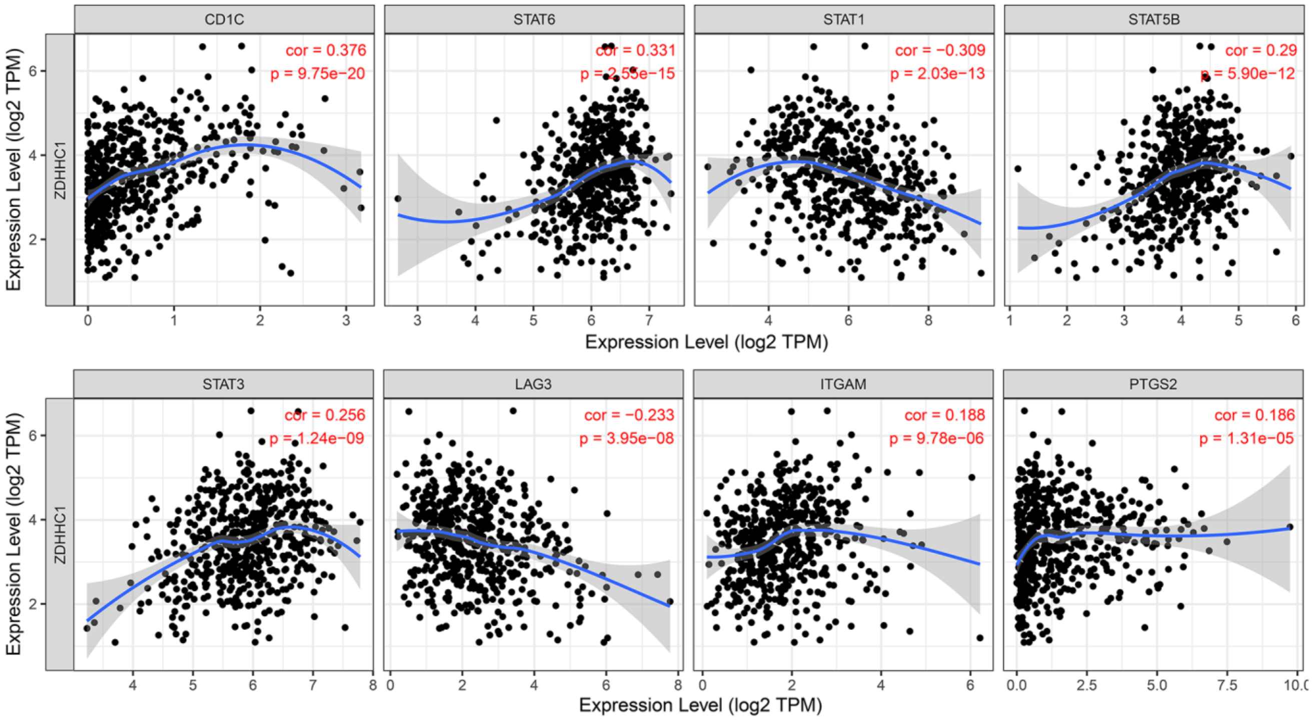

Supplementary Figure 4. Correlation between ZDHHC1 overexpression and immune cell markers.
