# Supplementary material for: Overexpression of zinc finger DHHC-type containing 1 is associated with poor prognosis and cancer cell growth and metastasis in uterine corpus endometrial carcinoma
Source: Aging (Albany NY). 2024 Jun 6;16(11):9784–812. doi: 10.18632/aging.205899 (PMC11210219; doi:10.18632/aging.205899)
Supplement: Supplementary Tables [file aging-16-205899-s002.docx]

**Supplementary Table 1. ZDHHC1 co-expressed genes.**

| Gene |  |  |  |  |  |
| --- | --- | --- | --- | --- | --- |
| DNAH3 | MSX1 | Z97653.2 | GRIN3B | FOXA2 | CFAP99 |
| ANKMY1 | MAP6 | PIGQ | LMF1 | RSPH3 | PITPNA |
| PIH1D2 | RBKS | MAP3K19 | GNA14 | SMDT1 | PTPA |
| FANK1 | KIAA0825 | LMX1A | ZSWIM7 | CYCSP34 | PIFO |
| KCNE1B | TP53AIP1 | CCDC191 | DNMBP | NWD1 | RIBC2 |
| STPG1 | DNAJB13 | ERGIC1 | CFAP65 | DHRS7B | C16orf71 |
| MINDY4 | LYRM9 | CFAP70 | AL390755.2 | TMEM63A | FAM189A2 |
| PPOX | GRIN1 | CFAP206 | AL138787.1 | NDRG2 | LDLRAD1 |
| TREH | EFCAB10 | AMPD3 | SLC47A1 | CUBN | TOM1 |
| HSDL2 | CRYL1 | CGNL1 | CCDC114 | IFT46 | TRPM4 |
| WFDC6 | PLA2G2C | NFIC | KIF9 | RFX2 | TMEM53 |
| TRAF3IP2 | KLLN | AURKA | KCNK6 | FAAH | HMG20B |
| CCDC188 | SMIM31 | ARMC3 | TRAPPC12 | C1orf194 | SCGB1D2 |
| AC027250.1 | CCDC60 | LRRC36 | ECT2L | FHAD1 | RRAD |
| KATNB1 | GAS2L2 | FRMPD2B | TRIM68 | B9D2 | IDUA |
| DCDC1 | CPNE2 | BAIAP3 | RAB36 | AC068587.2 | SERPINA6 |
| TMED6 | RPGRIP1L | USP43 | ANO1 | CRELD2 | LRRC37A5P |
| TTLL1 | CCDC157 | SCNN1G | TCTN1 | IGF2BP1 | HDHD3 |
| WDR90 | SLC25A35 | CFAP157 | DRC1 | FAM160B2 | SHISA6 |
| ZFHX2 | FUCA1 | CFAP46 | MAP3K15 | TMEM107 | TTC23L |
| ELAPOR1 | H2BC16P | CBY1 | LENG9 | WBP1L | CROCC2 |
| LRRC26 | LRRC49 | PODXL | CCDC173 | MAGI1 | GMPPB |
| TCTE1 | GLYATL2 | CYB5R1 | AC003072.1 | PHF7 | MYB |
| SORBS2 | GIPR | C11orf49 | C1orf158 | HGD | CFAP300 |
| C14orf93 | ZNF474 | GTF2F1 | CCDC17 | CAPS | SRARP |
| ZMYND10 | PRR29 | ZNF487 | WFS1 | L1CAM | PGR |
| ANKRD42 | VPS39 | FOXN4 | CROCC | FCSK | MYLK3 |
| TMEM232 | TIGD4 | ALG2 | ARMC2 | COMT | SCGB2A1 |
| HSPA1L | TTC8 | ZNF516 | C22orf15 | PAPSS1 | IFT22 |
| ALDH3A1 | MOK | ADH6 | ILDR1 | ECEL1P2 | TRPV4 |
| EFHC1 | C17orf50 | TEX264 | PLEKHH1 | PDLIM1 | FAM81B |
| ARMH1 | IRF6 | ADAT3 | MTSS2 | ZNF396 | EIF2D |
| EYA2 | CFAP74 | LRRC27 | ARFIP2 | VWA3A | C4orf47 |
| RSPH10B2 | STAP2 | LRWD1 | TNFAIP8L1 | TUBA4B | SLC43A1 |
| FAM166B | PCYT1B | SPG7 | NRTN | FBXO36 | TEX9 |
| CFAP73 | C16orf46 | ENTPD2 | STK33 | MORN5 | PPIL6 |
| CHMP1A | TTC26 | C7orf57 | WDR54 | DYNLRB2 | ARHGAP26 |
| RAB19 | ZMYND12 | COQ9 | SLC38A10 | PTCH1 | COG7 |
| IDNK | MYO5C | BEST4 | CARD10 | CYB5D2 | UBE2C |
| OR7E47P | FBXO15 | TMX4 | ARSD | RILPL2 | GNB5 |
| KNCN | PEX11G | MARCHF2 | TSNAXIP1 | MUC5B | ANKRD54 |
| EYA4 | AC092868.1 | CFAP221 | PBLD | NME9 | CHADL |
| MOB3A | LMNTD1 | LRP2BP | CFAP52 | CD164L2 | GLRB |
| C9orf152 | ERMN | DCAKD | DMBT1 | MB | MROH9 |
| SPATS1 | C9orf116 | CCNO | CASC1 | ALAD | A4GALT |
| TEKT2 | AC092718.3 | ZNF497 | REPIN1 | C11orf97 | MPC1L |
| PLAG1 | C4BPB | DENND6B | MRAP2 | ABHD17A | CCDC13 |
| ENTPD3 | HHAT | APOBEC4 | WDR49 | GHDC | TMEM210 |
| TTK | DNAJC4 | RPH3AL | ZC2HC1C | ARF1 | ABHD6 |
| RUNDC1 | SPACA9 | ENDOG | TMC3 | FAM181B | ARSA |
| TTC29 | SYTL1 | RSPH1 | NHLRC4 | ACSM1 | CFAP58 |
| LRGUK | FBXO31 | NXF3 | C5orf49 | STOML3 | GLYATL1 |
| ATP9B | ANKUB1 | WDR78 | C2orf50 | NPHP1 | DNAAF1 |
| CCDC113 | ZNF837 | GLB1L | C21orf58 | SLC44A4 | CCDC40 |
| CCDC74B | C2CD2L | C4BPA | HMGXB3 | PAX9 | C1orf189 |
| P2RX4 | SLC37A1 | KIF19 | PNPLA7 | CFAP53 | RIIAD1 |
| IQCE | CFAP100 | CFAP69 | CEL | CCDC96 | FICD |
| FBP1 | DNAAF3 | AC073439.1 | GORASP1 | FBXW9 | MYCBPAP |
| BICDL2 | ODF3B | MED11 | BTG4 | UFC1 | PFDN4 |
| CNGA4 | ERICH3 | LRRC10B | HNF1A | TTLL13P | SEC16A |
| C15orf65 | GPR135 | CCDC189 | AKAP14 | KPNA7 | LRRC43 |
| RNF135 | LRRC63 | CEP126 | GFOD2 | GCNT3 | EFCAB1 |
| CIBAR2 | CIB1 | WDR31 | UBXN6 | COLCA2 | LRP5L |
| DNALI1 | CCDC125 | MAK | NPDC1 | PTPN3 | CCDC74A |
| IFT140 | ATP2C2 | PLEKHS1 | DNAH10 | MDM2 | DCST2 |
| SCGB3A1 | ANKFN1 | ANAPC2 | ANKRD66 | SMPD3 | BRD3OS |
| CDC25B | LRTOMT | UBXN11 | CCDC30 | CCDC150 | COQ4 |
| TMEM41A | CCDC170 | FAM13C | BTBD2 | DTHD1 | SLC22A4 |
| MLPH | DNAI2 | PLIN3 | PPP1R36 | IFT122 | IQCK |
| RBM24 | NXNL2 | CFAP161 | MORN1 | RFX3 | FAM229B |
| ZFYVE28 | SLC5A1 | ANKDD1B | TMPRSS2 | FAM174B | MIER2 |
| CIRBP | G6PC3 | CCDC57 | TKFC | MID1IP1 | TEKT3 |
| TPPP3 | NME5 | ANG | FAM216B | RABL2B | SSXP10 |
| HHATL | TFF3 | MANSC1 | RN7SL250P | OVGP1 | NADSYN1 |
| TEKT1 | ENKUR | SLC40A1 | TMC5 | C6 | CRAT |
| ODF2 | IFT88 | GAS8 | FAM170A | RNF7P1 | AC005077.4 |
| STIM1 | SAT1 | C5AR2 | ZNF446 | DUOX1 | LINC00475 |
| CAPSL | NTN1 | RAET1E | ADGRG3 | MDH1B | BMP15 |
| ZNF213 | CDK20 | FAM221B | NUP62CL | MAPRE3 | ZNF19 |
| FAM81A | ARHGAP29 | SRD5A3 | TMEM254 | CC2D2A | CREB3L4 |
| PEMT | SPATA6 | FAM166C | BMPR1B | NLRX1 | C6orf52 |
| RSPH14 | SLC27A1 | TMEM220 | TTLL11 | ZBTB7A | DZIP3 |
| CUEDC1 | SPATA17 | ATAD2 | BBS12 | PALMD | UBXN10 |
| TRIM55 | COBL | TMEM114 | SELENOO | MORN2 | DNAH11 |
| CCDC153 | SAMD15 | ESR1 | CFAP57 | HID1 | TTC25 |
| TTC12 | PRKAB1 | P4HTM | FOXJ1 | PIK3R1 | CFAP251 |
| DNAH9 | CAPN6 | LCA5L | PPP1R32 | ZSWIM5 | CYB561 |
| TOGARAM2 | PACRG | RANBP10 | PKIG | WHRN | ZG16B |
| LRRC56 | TMEM9 | C20orf85 | IQCG | SPRYD3 | SIDT1 |
| TOR2A | RIBC1 | CCDC151 | SPEF2 | CCDC160 | AC004836.1 |
| WDR63 | TSKU | AK9 | EFHB | TMEM190 | OR7E36P |
| ABR | MCF2L | VWA5B2 | AP000769.1 | TLE5 | FAM47E |
| CCDC92 | BBS4 | TMC4 | STK11 | TCTEX1D4 | MZF1 |
| FAM183A | IQCH | DNAH7 | PLG | ZER1 | ATP5PBP5 |
| CHST6 | CCNE1 | HMGCL | EEF2 | LRRC6 | C10orf95 |
| TTLL9 | CTNNBIP1 | CFAP77 | UBBP4 | CFAP45 | VWA3B |
| SPPL2B | SAXO2 | DPCD | SMPD2 | PLPP2 | C2orf73 |
| FAM72D | CCDC65 | BBOF1 | LRRC46 | ANAPC4 | ZBBX |
| TMEM231 | FBXL8 | CCDC187 | IHH | C1QTNF8 | SHISA8 |
| POLQ | WWP2 | PZP | FAM149A | TMEM212 | BBS2 |
| IL20RA | B4GALNT3 | DRC3 | BBS5 | TMEM175 | RSPH4A |
| CENPA | NICN1 | PCSK4 | KNDC1 | KCNE1 | ACY3 |
| CCDC33 | SPDEF | AK7 | KIAA0556 | AKNA | TP73 |
| TGM3 | GSTK1 | DNAL4 | LRRC29 | TRIM3 | CDHR4 |
| SLC47A2 | GADD45G | MCIDAS | ZNF688 | WDR38 | LRRC23 |
| PLA2G10 | IFT43 | EXD3 | CDC20B | AADACP1 | DYDC1 |
| AC005041.1 | NEIL1 | IFT172 | CDHR3 | WDR34 | CERKL |
| AC013470.2 | FRMPD2 | LRRC73 | TMEM101 | COL28A1 | DNAJA4 |
| RNF183 | JHY | SGSM3 | KDM4B | GSTA3 | FN3K |
| SPATA18 | TBC1D30 | ERICH2 | MORN3 | OMG | PDCD5 |
| TTLL10 | SCAMP4 | ST6GALNAC2 | ITPRID1 | LRRC71 | DRC7 |
| DZIP1L | NANS | TRADD | LRRC74B | DACT2 | ST6GALNAC1 |
| CFAP299 | MTHFSD | ACAD10 | TTC16 | NEK5 | EFCAB2 |
| KLHDC9 | DNAI1 | MS4A8 | NEK11 | SMIM14 | SNTN |
| ENKD1 | KIF2C | C6orf118 | VMAC | CNDP2 | CABIN1 |
| CDKN1A | B9D1 | DYNLT1 | NUDT18 | DNAAF6 | DNAH12 |
| SCGB2A2 | EPPIN | AK8 | SNX10 | SDR42E2 | CCDC78 |
| PRMT7 | C10orf88B | AC099521.3 | TEKT4 | ASRGL1 | TMEM132A |
| KCNRG | SPEF1 | LNX1 | TTC38 | SLC22A5 | PMM1 |
| TTC21A | TCTN2 | B4GALT1 | GOLM1 | SLC52A1 | CREB3L1 |
| TBC1D8 | DECR2 | FBXW4 | CFAP61 | IQCD | C11orf16 |
| NEK10 | Y_RNA | CPXCR1 | AGR3 | ANKK1 | MPST |
| STX18 | KIF6 | ARMC4 | EFCAB12 | GREB1 | ENO4 |
| GPR19 | CATSPERD | ELP3 | HERC5 | STAR | SPRY3 |
| PTCH2 | TSPAN11 | DCDC2B | AC069366.1 | GET1 | AC007906.2 |
| TNFSF13 | AGBL2 | DNAAF4 | RALGDS | WDR93 | DUSP18 |
| MARCHF10 | SCNN1B | MKS1 | CHCHD7 | NDC80 | DNAH5 |
| TPX2 | RAB11B | ROPN1L | C11orf52 | BORCS6 | DLEC1 |
| HES5 | STMND1 | C9orf24 | CALML4 | RARA | WDR60 |
| XG | PLEKHG7 | FDXR | AZU1 | IL5RA | ORAOV1P1 |
| SERINC2 | DNAL1 | EPPIN-WFDC6 | AC010255.3 | TJP3 | CRY2 |
| KRT40 | CABCOCO1 | C17orf97 | SERPINA4 | DNAH6 | MYL12BP2 |
| SPA17 | SPATA33 | ARHGEF38 | SLC23A1 | IQUB | SMIM6 |
| CFAP43 | NT5E | ADGB | MAP1A | PRM2 | SERPINA11 |
| ECEL1P1 | PIGR | LY6G5C | ADAMTS8 | TUBB4B | DIS3L2 |
| FAM174A | DGLUCY | ZNF282 | OSCP1 | PLPPR3 | CATIP |
| MUC13 | LRRC18 | PPP1R42 | SPAG8 | GPR108 | SRD5A2 |
| EFCAB6 | DNAJC22 | PAM |  |  |  |

**Supplementary Table 2. Functions and molecular pathways of ZDHHC1 co-expressed genes.**

| **Category** | **Term** | **Count** | **P value** |
| --- | --- | --- | --- |
| BP | GO:0060271~cilium assembly | 56 | 4.83E-34 |
| BP | GO:0003341~cilium movement | 24 | 3.70E-22 |
| BP | GO:0030317~flagellated sperm motility | 25 | 4.29E-16 |
| BP | GO:0035082~axoneme assembly | 13 | 8.26E-12 |
| BP | GO:0044458~motile cilium assembly | 13 | 1.40E-11 |
| BP | GO:0007288~sperm axoneme assembly | 13 | 1.40E-11 |
| BP | GO:0007018~microtubule-based movement | 18 | 1.78E-09 |
| BP | GO:0003351~epithelial cilium movement | 10 | 3.15E-09 |
| BP | GO:0007368~determination of left/right symmetry | 15 | 6.19E-09 |
| BP | GO:1905515~non-motile cilium assembly | 14 | 1.54E-08 |
| BP | GO:0036159~inner dynein arm assembly | 9 | 1.58E-08 |
| BP | GO:0030030~cell projection organization | 14 | 8.25E-08 |
| BP | GO:0036158~outer dynein arm assembly | 9 | 2.69E-07 |
| BP | GO:0042073~intraciliary transport | 10 | 5.04E-07 |
| BP | GO:0060294~cilium movement involved in cell motility | 7 | 1.25E-06 |
| BP | GO:0070286~axonemal dynein complex assembly | 7 | 3.46E-06 |
| BP | GO:0060285~cilium-dependent cell motility | 7 | 5.39E-06 |
| BP | GO:0044782~cilium organization | 8 | 7.97E-06 |
| BP | GO:0007224~smoothened signaling pathway | 13 | 9.40E-06 |
| BP | GO:0061512~protein localization to cilium | 9 | 1.16E-05 |
| BP | GO:0051649~establishment of localization in cell | 14 | 1.18E-05 |
| BP | GO:0021532~neural tube patterning | 5 | 1.32E-05 |
| BP | GO:0060287~epithelial cilium movement involved in determination of left/right asymmetry | 6 | 2.98E-05 |
| BP | GO:0003356~regulation of cilium beat frequency | 5 | 3.00E-05 |
| BP | GO:0060122~inner ear receptor stereocilium organization | 6 | 3.00E-04 |
| BP | GO:0007286~spermatid development | 11 | 6.76E-04 |
| BP | GO:0090660~cerebrospinal fluid circulation | 5 | 9.59E-04 |
| BP | GO:1904491~protein localization to ciliary transition zone | 4 | 0.001487735 |
| BP | GO:0035720~intraciliary anterograde transport | 5 | 0.00159112 |
| BP | GO:0007026~negative regulation of microtubule depolymerization | 6 | 0.002132465 |
| BP | GO:0060296~regulation of cilium beat frequency involved in ciliary motility | 4 | 0.003043077 |
| BP | GO:0001843~neural tube closure | 9 | 0.003931315 |
| BP | GO:0032402~melanosome transport | 5 | 0.004324165 |
| BP | GO:1990961~drug transmembrane export | 4 | 0.005326161 |
| BP | GO:0021670~lateral ventricle development | 4 | 0.005326161 |
| BP | GO:0006656~phosphatidylcholine biosynthetic process | 5 | 0.006948038 |
| BP | GO:0031145~anaphase-promoting complex-dependent catabolic process | 5 | 0.006948038 |
| BP | GO:0042733~embryonic digit morphogenesis | 7 | 0.007001134 |
| BP | GO:0035721~intraciliary retrograde transport | 4 | 0.008414325 |
| BP | GO:0071468~cellular response to acidic pH | 4 | 0.008414325 |
| BP | GO:1903251~multi-ciliated epithelial cell differentiation | 3 | 0.009057563 |
| BP | GO:0009609~response to symbiotic bacterium | 3 | 0.009057563 |
| BP | GO:0045494~photoreceptor cell maintenance | 6 | 0.011391418 |
| BP | GO:1990403~embryonic brain development | 4 | 0.012362301 |
| BP | GO:0006165~nucleoside diphosphate phosphorylation | 4 | 0.012362301 |
| BP | GO:0034976~response to endoplasmic reticulum stress | 8 | 0.015585666 |
| BP | GO:0001947~heart looping | 7 | 0.017675872 |
| BP | GO:0033504~floor plate development | 3 | 0.018250469 |
| BP | GO:0033365~protein localization to organelle | 4 | 0.019968826 |
| BP | GO:0031018~endocrine pancreas development | 4 | 0.02296186 |
| BP | GO:0008589~regulation of smoothened signaling pathway | 4 | 0.02296186 |
| BP | GO:0042048~olfactory behavior | 3 | 0.023838047 |
| BP | GO:0060972~left/right pattern formation | 3 | 0.023838047 |
| BP | GO:0000226~microtubule cytoskeleton organization | 10 | 0.02413975 |
| BP | GO:0060173~limb development | 5 | 0.027107345 |
| BP | GO:0006886~intracellular protein transport | 18 | 0.028315945 |
| BP | GO:0032091~negative regulation of protein binding | 7 | 0.02904995 |
| BP | GO:0007052~mitotic spindle organization | 6 | 0.03170567 |
| BP | GO:0098719~sodium ion import across plasma membrane | 4 | 0.033315424 |
| BP | GO:0007339~binding of sperm to zona pellucida | 5 | 0.034898678 |
| BP | GO:0070254~mucus secretion | 3 | 0.036771717 |
| BP | GO:0070542~response to fatty acid | 4 | 0.037220353 |
| BP | GO:0042493~response to drug | 16 | 0.040712835 |
| BP | GO:0007507~heart development | 13 | 0.041349671 |
| BP | GO:0043010~camera-type eye development | 5 | 0.043857659 |
| BP | GO:0034504~protein localization to nucleus | 5 | 0.043857659 |
| BP | GO:0010669~epithelial structure maintenance | 3 | 0.04403471 |
| BP | GO:0050891~multicellular organismal water homeostasis | 3 | 0.04403471 |
| BP | GO:1902017~regulation of cilium assembly | 4 | 0.045696482 |
| BP | GO:0007346~regulation of mitotic cell cycle | 6 | 0.045855941 |
| BP | GO:0090630~activation of GTPase activity | 9 | 0.046460934 |
| BP | GO:0032880~regulation of protein localization | 7 | 0.046629405 |
| CC | GO:0005929~cilium | 59 | 3.89E-34 |
| CC | GO:0031514~motile cilium | 42 | 7.42E-32 |
| CC | GO:0036064~ciliary basal body | 47 | 5.06E-30 |
| CC | GO:0005930~axoneme | 38 | 7.29E-27 |
| CC | GO:0036126~sperm flagellum | 24 | 6.14E-18 |
| CC | GO:0097729~9+2 motile cilium | 15 | 3.52E-17 |
| CC | GO:0005879~axonemal microtubule | 17 | 4.32E-15 |
| CC | GO:0005874~microtubule | 41 | 3.76E-13 |
| CC | GO:0030286~dynein complex | 11 | 8.56E-09 |
| CC | GO:0036038~MKS complex | 8 | 4.98E-08 |
| CC | GO:0005813~centrosome | 42 | 5.33E-07 |
| CC | GO:0005737~cytoplasm | 236 | 8.35E-07 |
| CC | GO:0005814~centriole | 19 | 4.14E-06 |
| CC | GO:0005858~axonemal dynein complex | 7 | 4.21E-06 |
| CC | GO:0036157~outer dynein arm | 6 | 1.33E-05 |
| CC | GO:0097546~ciliary base | 10 | 1.90E-05 |
| CC | GO:0097542~ciliary tip | 10 | 1.90E-05 |
| CC | GO:0002177~manchette | 7 | 2.05E-05 |
| CC | GO:0005815~microtubule organizing center | 17 | 6.04E-05 |
| CC | GO:0035869~ciliary transition zone | 8 | 1.05E-04 |
| CC | GO:0032391~photoreceptor connecting cilium | 8 | 2.54E-04 |
| CC | GO:0097728~9+0 motile cilium | 4 | 3.15E-04 |
| CC | GO:0005856~cytoskeleton | 34 | 3.55E-04 |
| CC | GO:0060170~ciliary membrane | 9 | 7.16E-04 |
| CC | GO:0030991~intraciliary transport particle A | 4 | 0.00164096 |
| CC | GO:0030992~intraciliary transport particle B | 5 | 0.001803155 |
| CC | GO:0015630~microtubule cytoskeleton | 16 | 0.001821676 |
| CC | GO:0005881~cytoplasmic microtubule | 8 | 0.002251909 |
| CC | GO:0034464~BBSome | 4 | 0.002402834 |
| CC | GO:0097730~non-motile cilium | 6 | 0.002473191 |
| CC | GO:0001669~acrosomal vesicle | 11 | 0.003737642 |
| CC | GO:0097228~sperm principal piece | 6 | 0.003805472 |
| CC | GO:0097225~sperm midpiece | 7 | 0.004658107 |
| CC | GO:0016324~apical plasma membrane | 22 | 0.01048471 |
| CC | GO:0070062~extracellular exosome | 91 | 0.012332017 |
| CC | GO:0034706~sodium channel complex | 3 | 0.01420227 |
| CC | GO:0043025~neuronal cell body | 22 | 0.023780737 |
| CC | GO:0031232~extrinsic component of external side of plasma membrane | 3 | 0.031975205 |
| CC | GO:0034451~centriolar satellite | 9 | 0.035154304 |
| CC | GO:0005680~anaphase-promoting complex | 4 | 0.036310855 |
| CC | GO:0030175~filopodium | 7 | 0.046102475 |
| CC | GO:0005819~spindle | 10 | 0.049834391 |
| MF | GO:0045505~dynein intermediate chain binding | 11 | 2.21E-07 |
| MF | GO:0008569~ATP-dependent microtubule motor activity, minus-end-directed | 8 | 1.17E-06 |
| MF | GO:0051959~dynein light intermediate chain binding | 8 | 2.50E-05 |
| MF | GO:0005515~protein binding | 468 | 3.36E-04 |
| MF | GO:0008017~microtubule binding | 21 | 8.17E-04 |
| MF | GO:0003777~microtubule motor activity | 9 | 9.24E-04 |
| MF | GO:0043014~alpha-tubulin binding | 8 | 0.001118066 |
| MF | GO:0048487~beta-tubulin binding | 7 | 0.002800339 |
| MF | GO:0008158~hedgehog receptor activity | 3 | 0.003389265 |
| MF | GO:0004550~nucleoside diphosphate kinase activity | 5 | 0.004164702 |
| MF | GO:0030332~cyclin binding | 6 | 0.005515168 |
| MF | GO:0015631~tubulin binding | 8 | 0.005916168 |
| MF | GO:0070840~dynein complex binding | 5 | 0.008203612 |
| MF | GO:0008381~mechanically-gated ion channel activity | 4 | 0.008687413 |
| MF | GO:0045504~dynein heavy chain binding | 4 | 0.010780728 |
| MF | GO:0005524~ATP binding | 68 | 0.022880396 |
| MF | GO:0004017~adenylate kinase activity | 3 | 0.035504949 |
| MF | GO:0019211~phosphatase activator activity | 3 | 0.035504949 |
| MF | GO:0070740~tubulin-glutamic acid ligase activity | 3 | 0.043397855 |
| MF | GO:0004620~phospholipase activity | 4 | 0.046678642 |
| KEGG | hsa01100: Metabolic pathways | 57 | 6.24E-04 |
| KEGG | hsa05016: Huntington disease | 17 | 0.002916623 |
| KEGG | hsa00051: Fructose and mannose metabolism | 5 | 0.00778237 |
| KEGG | hsa04152: AMPK signaling pathway | 9 | 0.008778841 |
| KEGG | hsa05215: Prostate cancer | 8 | 0.008825632 |
| KEGG | hsa00350: Tyrosine metabolism | 5 | 0.010592786 |
| KEGG | hsa04340: Hedgehog signaling pathway | 6 | 0.010969036 |
| KEGG | hsa05014: Amyotrophic lateral sclerosis | 17 | 0.014692728 |
| KEGG | hsa01232: Nucleotide metabolism | 7 | 0.016529724 |
| KEGG | hsa04915: Estrogen signaling pathway | 9 | 0.018349784 |
| KEGG | hsa04975: Fat digestion and absorption | 5 | 0.019485351 |
| KEGG | hsa00520: Amino sugar and nucleotide sugar metabolism | 5 | 0.029942492 |
| KEGG | hsa05231: Choline metabolism in cancer | 7 | 0.030849019 |
| KEGG | hsa04115: p53 signaling pathway | 6 | 0.031142413 |
| KEGG | hsa04110: Cell cycle | 8 | 0.032536067 |
| KEGG | hsa00230: Purine metabolism | 8 | 0.035011263 |
| KEGG | hsa04931: Insulin resistance | 7 | 0.046170837 |
